# Efficacy and Safety of EGF/EGFR Vaccines in EGFR‐Driven Solid Tumors: A Systematic Review and Meta‐Analysis of Controlled and Single‐Arm Studies

**DOI:** 10.1002/cam4.71295

**Published:** 2025-10-09

**Authors:** Fei Chen, Ling Bai, Jiuwei Cui

**Affiliations:** ^1^ Cancer Center The First Hospital of Jilin University Changchun China; ^2^ Department of Pathology Duke University School of Medicine Durham North Carolina USA

**Keywords:** combination immunotherapy, EGF vaccine, EGFR vaccine, EGFR‐driven solid tumors, meta‐analysis

## Abstract

**Background:**

Despite multiple clinical trials, the benefits and safety of epidermal growth factor (EGF)/EGF receptor (EGFR) vaccines in EGFR‐driven solid tumors remain unclear due to small sample sizes and heterogeneous study designs. This systematic review and meta‐analysis aimed to evaluate their efficacy and safety.

**Methods:**

We conducted pairwise and single‐arm meta‐analyses following PRISMA guidelines (PROSPERO: CRD420251026774). Searches in PubMed, Embase, and Cochrane Library identified 26 trials (2701 participants). The primary endpoint was overall survival (OS), with secondary analyses of progression‐free survival (PFS), objective response rate (ORR), disease control rate (DCR), and treatment‐related adverse events (TRAEs). Statistical analyses were performed using R software, with fixed or random‐effects models per heterogeneity (*I* ^2^).

**Results:**

Compared with best supportive care, vaccine monotherapy significantly improved long‐term OS in NSCLC and GBM (3‐year OR = 2.16, 5‐year OR = 3.20) and prolonged median OS in NSCLC (HR = 0.76). In single‐arm studies, NSCLC patients receiving vaccine monotherapy had a 1‐year OS of 64% (75% in first‐line maintenance), with an ORR of 2% and DCR of 31%. For GBM, vaccine combination therapy improved 3‐year OS (OR = 2.42) and 2‐year PFS (OR = 1.63) versus standard therapy. Single‐arm combination analyses showed an overall 1‐year OS of 84%, ORR of 42%, and DCR of 87%, while NSCLC first‐line combination achieved a 1‐year OS of 85% and DCR of 89%. Notably, EGFR‐mutant NSCLC patients had a pooled ORR of 65% and DCR of 98%. Common TRAEs were grade 1–2 (injection site reactions, fever, headache, and vomiting), and combination therapy had no new or severe toxicities.

**Conclusions:**

EGF/EGFR vaccines may improve survival in EGFR‐driven solid tumors, particularly NSCLC and GBM. Monotherapy enables long‐term disease control, and combination therapy enhances efficacy without added toxicity, supporting further clinical validation.

**Trial Registration:**

PROSPERO CRD420251026774

## Graphical abstract

This systematic review and meta‐analysis suggest that EGF/EGFR vaccines may improve survival in EGFR‐driven solid tumors (notably NSCLC/GBM), with monotherapy providing long‐term disease control and combination therapy showing a potential to extend survival without added toxicity, supporting their promise for future clinical application pending further validation.

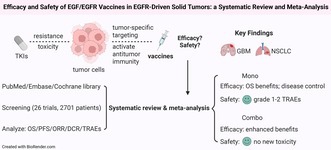

**Abbreviations**
AEs: adverse events
BLyS: B lymphocyte stimulator
CIs: confidence intervals
CRC: colorectal cancer
DCR: disease control rate
DLTs: dose‐limiting toxicities
EGF: epidermal growth factor
EGFR: epidermal growth factor receptor
EGFRvIII: epidermal growth factor receptor variant III
GAR: good antibody responders
GBM: glioblastoma
HRs: hazard ratios
ICI: immune checkpoint inhibitor
irRECIST: immune‐related Response Evaluation Criteria in Solid Tumors
ITT: intent‐to‐treat
LC: lung cancer
MINORS: Methodological Index for Non‐randomized Studies tool
MRD: minimal residual disease
NSCLC: non‐small cell lung cancer
ORR: objective response rate
ORs: odds ratios
OS: overall survival
PC: pancreatic cancer
PD‐L1: programmed death‐ligand 1
PFS: progression‐free survival
RCTs: randomized controlled trials
RECIST: Response Evaluation Criteria in Solid Tumors
SAEs: serious adverse events
sGAR: super good antibody responders
SRD: significant residual disease
TKIs: tyrosine kinase inhibitors
TMZ: temozolomide
TRAEs: treatment‐related adverse events

## 1 Introduction

Persistent activation of the epidermal growth factor (EGF)/epidermal growth factor receptor (EGFR) signaling axis drives progression in solid tumors such as non‐small cell lung cancer (NSCLC), glioblastoma (GBM), and hormone‐refractory prostate cancer [1, 2, 3, 4, 5, 6, 7, 8, 9, 10]. EGF, as the primary ligand for EGFR, binds to the receptor to initiate downstream signaling cascades that promote tumor cell proliferation, survival, angiogenesis, and metastasis, while EGFR dysregulation—via overexpression, mutation (e.g., EGFR variant III [EGFRvIII] in GBM), or ligand‐dependent activation—makes this axis a critical therapeutic target [4, 11]. However, current EGFR tyrosine kinase inhibitors (TKIs) or monoclonal antibodies often fail to control long‐term disease progression. This limitation arises from two key factors: acquired resistance (e.g., target mutations, bypass pathway activation) and the induction of immunosuppressive microenvironments. Both factors diminish host anti‐tumor immune responses and compromise durable efficacy [11, 12, 13, 14, 15, 16, 17, 18, 19, 20, 21, 22]. Moreover, this broad‐spectrum inhibition, while disrupting tumor signaling, also drives acquired resistance (via incomplete pathway blockade) and induces off‐target toxicities in EGFR‐expressing normal tissues, such as skin and gastrointestinal epithelia [23, 24, 25]. This limitation highlights the need for alternative strategies like vaccines.

Vaccines targeting EGF/EGFR axis components—including ligand (EGF) or receptor (EGFR/EGFRvIII)—represent a promising immunotherapeutic strategy. They disrupt oncogenic signaling via antibody‐mediated neutralization and enhance tumor immunogenicity through antigen‐specific T‐cell responses, addressing key limitations of traditional inhibitors [26, 27, 28, 29, 30]. Importantly, these vaccines exhibit tumor‐specific targeting via immune recognition of EGF/EGFR‐expressed antigens, offering a safer therapeutic window. By simultaneously disrupting oncogenic signaling and activating antitumor immunity, these vaccines address both resistance and immunosuppression—key limitations of traditional inhibitors. Moreover, this mechanistic distinction highlights potential safety advantages over traditional EGFR inhibitors, with preclinical studies demonstrating the overcoming of limitations of conventional anti‐EGFR therapies [31, 32].

Clinical trials have demonstrated favorable immunogenicity and safety profiles, with some studies reporting improved survival in NSCLC and GBM patients [33, 34, 35, 36, 37, 38, 39]. However, the phase III “ACT IV” trial (NCT01480479) showed that adding the EGFRvIII‐targeted vaccine rindopepimut to standard temozolomide (TMZ) therapy did not improve survival in newly diagnosed GBM patients [40]. Given the variability in trial results and limited evidence from individual studies, a comprehensive integration of multiple study datasets is necessary to accurately assess the antitumor efficacy and safety of EGF/EGFR vaccines.

To address this limitation, we performed a systematic review of clinical outcomes from randomized, non‐randomized controlled, and single‐arm trials evaluating EGF/EGFR‐targeted vaccines. Using meta‐analytic approaches, we systematically assessed their therapeutic efficacy and safety parameters. The objectives of this study were: (1) to clarify the survival benefit of vaccine monotherapy versus best supportive care; (2) to investigate efficacy across treatment lines; (3) to assess performance in lesion remission and disease control; (4) to verify synergistic effects of vaccine combination regimens; (5) to analyze combination therapy effectiveness across cancer types, treatment settings, and EGFR status; (6) to summarize the grades, types, and incidence rate of vaccine‐related common adverse events (AEs), as well as the safety profile of combination regimens. By integrating data from diverse studies, we seek to provide evidence‐based support for the application of EGF/EGFR vaccines in modern oncology practice—particularly for patients resistant or intolerant to traditional EGFR‐targeted therapies.

## 2 Materials and Methods

In this study, we performed a systematic review and meta‐analysis following PRISMA 2020 guidelines [41] to evaluate the efficacy and safety of EGF/EGFR vaccines across EGFR‐driven solid tumors. The complete PRISMA 2020 Checklist, which documents reporting compliance for both review and meta‐analytic methods, is provided as Table S1. The protocol was prospectively registered on the Prospero international prospective register of systematic reviews (registration number: CRD420251026774). Data integration included randomized controlled trials (RCTs), non‐randomized comparative studies, and single‐arm trials, with a focus on synthesizing outcomes including overall survival (OS), progression‐free survival (PFS), objective response rate (ORR), disease control rate (DCR), and treatment‐related adverse events (TRAEs).

### 2.1 Inclusion Criteria and Literature Search

Clinical trials were eligible for inclusion if they met all of the following criteria:

1. Evaluated therapeutic vaccines targeting the EGF/EGFR signaling axis (including ligands, receptors, or their variants);
2. Administered the vaccine either as monotherapy or combination therapy to patients with pathologically confirmed EGFR‐driven solid tumors;
3. Reported at least one predefined clinical endpoint (OS, PFS, ORR, DCR, or TRAEs);
4. Adopted a prospective study design (encompassing RCTs, non‐randomized controlled trials, or single‐arm studies).

Exclusion criteria included the following:

1. Studies involving non‐therapeutic vaccines (e.g., prophylactic vaccine studies);
2. Non‐comparative observational studies (e.g., case reports, retrospective studies that solely describe clinical characteristics);
3. Biomarker analysis studies (only reporting EGFR‐related biomarkers without providing efficacy data for the vaccine treatment);
4. Reports with missing data that rendered them unsuitable for analysis;
5. Studies with a sample size of less than 10;
6. Studies on personalized neoantigen peptide vaccines (vaccines not targeting the EGF/EGFR axis).

We conducted systematic searches across PubMed, Cochrane Library, and Embase from their inception through July 16, 2025, and supplemented the searches by manually screening relevant reviews and reference lists of included studies. The search strategy integrated modular concepts (‘neoplasms’, ‘EGFR’, ‘vaccines’) using controlled vocabulary (MeSH/Emtree) and free‐text terms, with full syntax provided in Table S2.

### 2.2 Data Extraction and Risk of Bias Assessment

We designed a spreadsheet using Excel to extract information from eligible studies. Two authors independently extracted trial characteristics, including trial name or author/year, phase, sample size, population demographics, intervention details, and main outcomes. For duplicate reports of the same study, the most updated data were extracted. Preferentially, data from the per‐protocol population and those subjected to central review were extracted. Median survival times were analyzed preferentially if both median and mean survival times were available. For survival rates, ORR, DCR, and AEs, we extracted the number of events and the total evaluable samples. TRAEs were prioritized when explicitly reported; otherwise, all‐cause AE data were recorded as documented in the source studies.

For survival time endpoints (including OS and PFS), we extracted data through two approaches. First, directly reported values—such as OS/PFS rates at specific time points and hazard ratios (HRs) with corresponding 95% confidence intervals (CIs)—were retrieved from the text, tables, or supplementary materials of the studies. Second, when these values were only presented in Kaplan–Meier curves, we digitized the data points (including time‐specific OS/PFS rates and raw survival data for HR calculation) using Engauge Digitizer 11.3. HRs and corresponding 95% CIs were derived from these digitized data using the method described by Jayne F. Tierney et al. [42].

Basic information and directly reported data were presented in Table 1, while digitized data were summarized in Table S3. Discrepancies in data extraction were resolved by the extractors through discussion, with cross‐referencing of original reports as needed.

**TABLE 1:** Main details of included studies.

[Article type] author, year (phase, trial name)	Sample size	Population	Intervention	Median age, year	Female, %	Median OS, month	Median PFS, month	OS rate, %	PFS rate, %	ORR, %	DCR, %	Grade ≥ 3 AEs %
*Controlled trials: Vaccine monotherapy vs. Best supportive care*												
**[J]Rodriguez, 2016 (III)** [33]	270	Advanced NSCLC, maintenance therapy after 1st‐line chemotherapy	EGF vaccine (CIMAvax‐EGF)	NR	34.10	12.43	NR	5y OS: 16.62%	NR	NR	NR	3.6
	135		best supportive care	NR	36.30	9.43	NR	5y OS: 6.2%	NR	NR	NR	0
**[J]Neninger, 2008 (II)** [43]	40	Advanced NSCLC, maintenance therapy after 1st‐line chemotherapy, or 2nd‐line	EGF vaccine (CIMAvax‐EGF)	58.5	25	6.47	NR	NR	NR	NR	NR	0
	40		best supportive care	54	27	5.33	NR	NR	NR	NR	NR	0
***[J]Sampson, 2010 (II, ACTIVATE)*** [35, 44]	18	Newly diagnosed EGFRvIII+ GBM, after gross total resection and chemoradiation	EGFRvIII vaccine (rindopepimut)	52	28	24.6	14.2	6m OS: 100%; 1y OS: 94%; 2y OS: 53%	6m PFS: 94%; 1y PFS: 28%; 2y PFS: 28%	NR	NR	0
	17		historical matched cohort without vaccine	59	52.9	15	6.3	6m OS: 94%; 1y OS: 71%; 2y OS: 6%	6m PFS: 59%; 1y PFS: 24%; 2y PFS: 6%	NR	NR	0
*Single‐arm trials: Vaccine monotherapy*												
**[J]Flores, 2023 (real‐world)** [45]	106	Advanced NSCLC, maintenance therapy after 1st‐line chemotherapy	EGF vaccine (CIMAvax‐EGF) 2.4mg per vaccination	NR	48.5	14.6	8.16	6m OS: 82.1%; 1y OS: 57.2%; 2y OS: 37.6%	6m PFS: 55.4%; 1y PFS: 36.4%; 2y PFS: 19.1%	12.3	36.8	0
**[CA]Kananathan, 2015** [46]	23	Advanced NSCLC, maintenance therapy after 1st‐line chemotherapy	EGF vaccine (CIMAvax‐EGF) 2.4mg per vaccination	55.18	30.40	21	NR	1y OS: 91%; 2y OS: 43%; 3y OS: 30%; 5y OS: 9%	NR	NR	NR	NR
**[J]Rodriguez, 2011 (III)** [47]	40	Advanced NSCLC, maintenance therapy after 1st‐line chemotherapy	EGF vaccine (CIMAvax‐EGF) 2.4mg per vaccination	NR	31.80	13.57	NR	2y OS: 34.2%	NR	NR	NR	0
**[J]Xing, 2018 (I)** [48]	21	Advanced NSCLC, maintenance therapy after 1st‐line chemotherapy	EGF vaccine (Hu‐rhEGF‐rP64k/Mont) 0.6‐2.4mg per vaccination	54	42.90	NR	NR	NR	NR	0	75	0
**[J]Rodriguez, 2011 (II)** [47]	40	Advanced NSCLC, maintenance therapy after 1st‐line chemotherapy or 2nd therapy	EGF vaccine (CIMAvax‐EGF) 0.6mg per vaccination	NR	25	6.47	NR	2y OS: 27.27%	NR	NR	NR	0
**[J]Ramos, 2006 (I)** [49]	21 22	Advanced NSCLC, completed 1st‐line therapy	EGF vaccine (CIMAvax‐EGF) 71ug per vaccination	59.6 (mean)	14	6.43	NR	NR	NR	NR	NR	0
			EGF vaccine (CIMAvax‐EGF) 142ug per vaccination	54.7 (mean)	23	8.4	NR	NR	NR	NR	NR	0
**[J]Ortiz, 2023 (IV)** [36]	741	Advanced NSCLC, ineligible for further onco‐specific treatment	EGF vaccine (CIMAvax‐EGF) 2.4mg per vaccination	65.22 (mean)	39.1	9.9	NR	1y OS: 42%; 2y OS: 19.5%	NR	NR	NR	0.4
**[J]Gonzalez, 2003 (I)** [50]	40	Advanced NSCLC, not amenable to any other modality of onco‐specific therapy	EGF vaccine (CIMAvax‐EGF)	62 (mean)	20	8.17	NR	NR	NR	0	30	0
**[CA]Liang, 2025** **I, ABOR2013–101‐GYF‐Part 1** [51]	20	Advanced NSCLC (with L858R/19del/T790M mutations)	EGFR mRNA vaccine (ABOR2013)	NR	NR	NR	NR	NR	NR	NR	NR	0
**[J]Xiong, 2021 (I)** [52]	16	Advanced solid tumors, ≥ 3rd‐line setting (13 LC, 2 CRC, 1 PC)	EGF vaccine (EGF‐CRM197) 0.4‐1.6mg per vaccination	59.54	25	(lung cancer) sGAR: not reached; GAR: 10.67	(lung cancer) sGAR: 4.83; GAR: 2.1	NR	NR	0	0	0
*Controlled trials: Vaccine combination therapy vs. onco‐specific standard therapy*												
**[J]Weller, 2017 (III, ACT IV)** [40]	371	Newly diagnosed EGFRvIII + GBM, adjuvant therapy after maximal surgical resection and chemoradiation	EGFRvIII vaccine (rindopepimut 500 μg) + TMZ	59	32	17.4	7.1	NR	NR	15	NR	43.1^a^
	374		KLH control + TMZ	58	39	17.4	5.6	NR	NR	15	NR	44.6^a^
***[J]Sampson, 2011 (II, ACT II)*** [39]	22	Newly diagnosed EGFRvIII + GBM, adjuvant therapy after maximal surgical resection and chemoradiation	EGFRvIII vaccine (rindopepimut) + TMZ	57 (mean)	NR	23.6	15.2	6m OS: 100%; 1y OS: 100%; 2y OS: 44.4%	6m PFS: 95.5%; 1y PFS: 63.6%; 2y PFS: 20.1%	NR	NR	18.2
	17		Historical matched cohort without vaccine	59	NR	15	6.3	6m OS: 94.1%; 1y OS: 70.6%; 2y OS: 5.9%	6m PFS: 58.8%; 1y PFS: 23.5%; 2y PFS: 5.9%	NR	NR	NR
**[J]Reardon, 2020 (II, ReACT)** [34]	36	Relapsed EGFRvIII + GBM	EGFRvIII vaccine (rindopepimut 500 μg) + bevacizumab	59	47.00	NR	3.7	2y OS: 20%	6m PFS: 28%	30	NR	NR
	37		KLH control + bevacizumab	55	41.00	NR	3.7	2y OS: 3%	6m PFS: 16%	18	NR	NR
[CA]Ills, 2010 (II) [53]	NR	Patients with hormone‐refractory prostate cancer	EGF vaccine (CIMAvax EGF)‐chemotherapy‐vaccine	NR	NR	17.1	NR	2y OS: 26.59%; 3y OS: 22.79%	NR	NR	NR	NR
	NR		Chemotherapy	NR	NR	10.73	NR	2y OS: 17.44%; 3y OS: 13.95%	NR	NR	NR	NR
*Single‐arm trials: Vaccine combination therapy*												
**[J]Rodríguez, 2022 (Ib, EPICAL)** [54]	23	Advanced 1st‐line EGFR‐mut NSCLC	EGF vaccine (hu‐recEGF/recP64k 2.4mg per vaccination) + afatinib	67	69.60	26.9	17.5	NR	NR	78.30	95.70	30
[CA]Neninger, 2019 (I) [55]	30	Advanced 1st‐line NSCLC	EGF vaccine (CIMAvax‐EGF 2.4mg per vaccination) + chemotherapy	NR	NR	9.4	NR	NR	NR	NR	NR	NR
**[CA]Andric, 2018 (II)** [56]	32	Advanced 1st‐line NSCLC	EGF vaccine (CIMAvax‐EGF) + chemotherapy	62.7 (mean)	50	NR	5.8 (mean)	NR	NR	18.75	84.38	NR
**[J]Neninger, 2009 (I)** [57]	20	Advanced 1st‐line NSCLC	EGF vaccine (CIMAvax‐EGF) + chemotherapy	58	20	12.8	8.4	1y OS: 70%	NR	35	85	NR
**[J]Suárez, 2022** [58]	18	Advanced NSCLC, after 1st‐line platinum‐based chemotherapy (94.4% 1st maintenance, 5.6% 2nd)	EGF vaccine (CIMAvax‐EGF 2.4 mg per vaccination) + thymic polypeptide fraction	62.64 (mean)	33.33	16.09	9.43	6m OS: 89%;1y OS: 66%	6m PFS: 72%;1y PFS: 33%	NR	NR	0
**[J]Evans, 2022 (I)** [38]	13	Advanced 2nd‐line NSCLC	EGF vaccine (CIMAvax‐EGF 1.2 or 2.4mg per vaccination) + nivolumab	63	69	18.3	NR	NR	NR	33	50	7.7
[CA]Liang, 2025 (I, ABOR2013‐101‐GYF)‐Part 2 [51]	10	Advanced NSCLC (with L858R/19del/T790M mutations)	EGFR mRNA vaccine (ABOR2013) + sintilimab	NR	NR	NR	NR	NR	NR	NR	NR	10
**[CA]Wang, 2024** [37]	11	Advanced NSCLC resistant to EGFR‐TKI therapy	EGFR vaccine + tislelizumab + chemotherapy	NR	NR	NR	NR	NR	NR	45.45	100	0
**[J]Schuster, 2015 (II, ACT III)** [44]	65	Newly diagnosed EGFRvIII+ GBM, after gross total resection and chemoradiation	EGFRvIII vaccine (rindopepimut 500μg) + TMZ	56	49	21.8	9.2	3y OS: 26%	5.5m PFS: 66%	NR	NR	NR
**[J]Sampson, 2009 (I)** [59]	12	Newly diagnosed GBM, after gross total resection and chemoradiation	EGFRvIII vaccine (rindopepimut 500μg) pulsed DC	42	33.33	18.7	6.8	6m OS: 92%; 1y OS: 83%; 2y OS: 42%	6m PFS: 58%; 1y PFS: 17%	NR	NR	NR

*Note:* Studies that are included in both the systematic review and the meta‐analysis are indicated in bold, while those included only in the systematic review are not bolded. Non‐randomized comparative studies are indicated *in italic*.

Abbreviations: CRC, colorectal cancer; GAR, good antibody responders; KLH, keyhole limpet hemocyanin; LC, lung cancer; M, month; NR, not reported; PC, pancreatic cancer; sGAR, super good antibody responders; y, year.

^a^ All‐cause AEs.

Two investigators independently assessed the risk of bias for included studies: the Cochrane Risk of Bias Tool was used for RCTs, and the Methodological Index for Non‐randomized Studies (MINORS) for non‐randomized comparative and single‐arm trials. Any discrepancies were addressed via group deliberation.

### 2.3 Statistical Analyses

OS was designated as the primary outcome to specifically reflect the long‐term efficacy of EGF/EGFR vaccines, as their antitumor effects may manifest sustainably or with delays over time. Secondary outcomes included PFS, ORR, DCR, and incidence of TRAEs.

#### 2.3.1 Primary Analyses

For controlled trials, direct pairwise comparisons were performed. Specifically, HRs were calculated for survival time comparisons (e.g., median OS/PFS), and odds ratios (ORs) were calculated for time‐point survival rates, ORR, DCR, and TRAE incidence. For single‐arm studies, pooled event proportions were derived. All these metrics (HRs, ORs, proportions) were presented with 95% CIs. All analyses were conducted using R software (version 4.4.2). Meta‐analysis results, including effect estimates (HRs/ORs) and pooled event proportions (with their corresponding 95% CIs) for all outcomes, were visualized using forest plots to depict study‐level data and pooled effects.

Heterogeneity was assessed using *I* ^2^ statistics and *Q*‐test. Significant heterogeneity was defined as *I* ^2^ > 50% and *Q*‐test *p* < 0.1, in which case random‐effects models were applied; otherwise, common‐effect (fixed‐effect) models were used. For studies with zero events, a continuity correction method with a correction factor of 0.5 was implemented [60, 61].

#### 2.3.2 Subgroup Analyses

To explore potential sources of heterogeneity and identify preferential populations for EGF/EGFR vaccines, we pre‐specified subgroup analyses based on clinical relevance and data availability. The predefined subgroup variables included cancer type, vaccine target, treatment line, EGFR status, and vaccine name.

Given that GBM uses EGFRvIII‐targeted vaccines and NSCLC uses EGF/EGFR‐targeted vaccines, treatment line stratification inherently incorporates cancer type and target differences. For instance, adjuvant GBM studies exclusively use EGFRvIII‐targeted vaccines, while first‐line NSCLC studies utilize EGF‐targeted vaccines. Thus, treatment line stratification naturally distinguishes these cancer type and target‐specific differences, as visually confirmed in the forest plots. Therefore, we primarily used treatment line as the subgroup in the meta‐analysis, with results presented in forest plots that explicitly label each study's cancer type, vaccine target, and treatment line. This allows simultaneous interpretation of cancer type and target subgroups despite the primary stratification by treatment line.

In the DCR analysis of vaccine combination therapy, two single‐arm studies explicitly reported EGFR mutations, while the other three had wild‐type EGFR or unknown status, prompting us to conduct subgroup analyses based on EGFR status.

In addition, subgroup stratification was further performed using vaccine names to compare the incidence of TRAEs across different vaccine types, aiming to identify potential safety differences between vaccine formulations.

#### 2.3.3 Sensitivity Analyses

To validate the robustness of pooled results and explore the potential impact of individual studies as well as sources of heterogeneity, we performed sensitivity analyses using the leave‐one‐out method for outcomes with three or more included studies. Specifically, each study was sequentially excluded, and the pooled effect size with 95% CIs was recalculated, with concurrent monitoring of changes in the *I* ^2^ statistic for heterogeneity. For comparative analyses, robustness was determined by whether the 95% CI of the effect size crossed the null value of 1. For proportional meta‐analyses, robustness was defined using prespecified thresholds: a > 15% change in effect size or a > 25% reduction in heterogeneity, balancing statistical significance and clinical relevance [60, 62, 63].

## 3 Results

### 3.1 Study Selection and Characteristics

**FIGURE 1:**
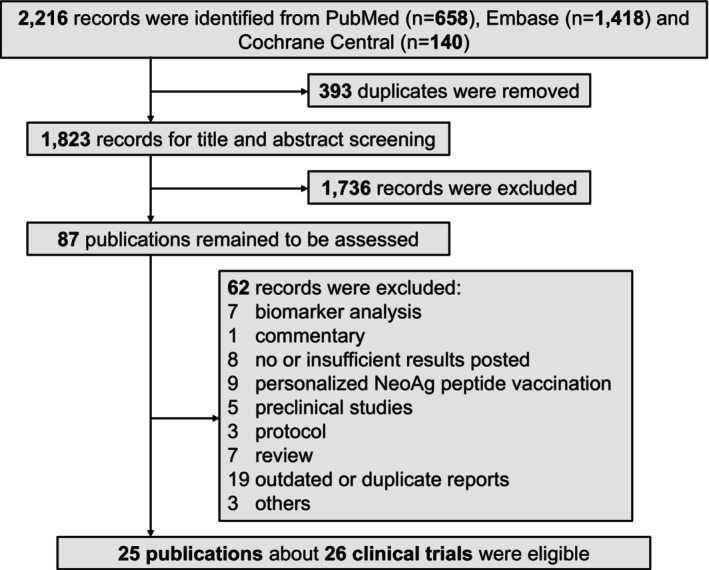
Flowchart of the study selection process.

The initial literature search identified 2216 records. After removing 393 duplicates, we screened 1823 titles/abstracts and assessed 87 studies. Ultimately, 25 citations involving 26 clinical trials (enrolling a total of 2701 patients) met the inclusion criteria (Figure 1).

The included studies comprised 5 RCTs, 2 non‐randomized comparative trials, and 19 single‐arm studies, with 18 in NSCLC, 6 in GBM, 1 in prostate cancer, and 1 in mixed solid tumors (lung cancer [LC], colorectal cancer [CRC], and pancreatic cancer [PC]). Based on study design and intervention, the included studies were classified into four categories:

1. Controlled trials comparing vaccine against best supportive care: 2 NSCLC trials (EGF vaccine) and 1 GBM trial (EGFRvIII vaccine) [33, 35, 43, 44];
2. Single‐arm trials of vaccine monotherapy: 9 studies for NSCLC (8 of EGF vaccine, 1 of EGFR vaccine) and 1 study for mixed solid tumors (cases of LC, CRC, and PC) (EGF vaccine) [36, 45, 46, 47, 48, 49, 50, 51, 52];
3. Controlled trials of vaccine combination therapy versus tumor‐specific standard treatment: 3 GBM studies (EGFRvIII vaccine) and 1 prostate cancer study (EGF vaccine) [34, 39, 40, 53];
4. Single‐arm studies of vaccine combination therapy: 8 NSCLC trials (6 of EGF vaccine, 2 of EGFR vaccine) and 2 GBM trials (EGFRvIII vaccine) [37, 38, 44, 51, 54, 55, 56, 57, 58, 59].

Among these, several studies required additional clarification due to their specific design features or data handling considerations. The phase I trial by Liang et al. (ABOR2013‐101‐GYF, ChiCTR2300071001) included two dose‐escalation arms: EGFR vaccine monotherapy (part 1) and EGFR vaccine combined with sintilimab (an immune checkpoint inhibitor [ICI]) (part 2) [51]; thus this trial was included in both categories 2 and 4. Rodriguez et al. (2011) reported both a phase II and a phase III trial within a single publication [47]. Sampson et al. (2011) compared two experimental arms (EGFRvIII vaccine plus standard‐dose or dose‐intensified TMZ) against one historical TMZ control group [39]; since PFS and OS did not differ significantly between TMZ regimens, pooled data were extracted. The 3‐ to 5‐year OS data of the ACTIVATE study (Sampson et al. 2010) were updated in the “ACT III” trial report by Schuster et al. (2015) [44].

Key details reported in eligible studies are summarized in Table 1, with directly extracted data (e.g., baseline characteristics, explicitly reported outcomes) presented therein. Data digitized from survival curves using Engauge Digitizer 11.3 (including time‐specific survival rates and calculated HRs) are available in Table S3. Of these, 24 studies (bolded in Table 1) were included in both the systematic review and meta‐analysis, while 2 studies (non‐bolded in Table 1) contributed only to the systematic review. EGF vaccines were evaluated in 18 clinical trials, including 16 trials for NSCLC, 1 trial for prostate cancer, and 1 trial for mixed cancer patients. EGFR vaccines were examined in 2 clinical trials targeting NSCLC. The EGFRvIII vaccine was investigated in 6 clinical trials focusing on GBM.

### 3.2 Evaluation of Study Quality

Beyond descriptive characteristics, we further evaluated the methodological robustness of included evidence through standardized bias assessment tools. The methodological quality assessment revealed that all single‐arm trials demonstrated low risk of bias, characterized by clearly defined objectives, explicit inclusion/exclusion criteria, appropriate outcome measures, and adequate follow‐up periods (Figure S1). Among the controlled studies, the two non‐randomized comparative trials included in the meta‐analysis were rated as low risk. However, the RCT conducted by Neninger et al. (2008) was at high risk of bias owing to inadequate allocation concealment and lack of blinding. Similarly, the study by IIIs et al. (2010, included only in the systematic review) was classified as high‐risk due to unblinded design. The remaining studies maintained high methodological quality throughout their design and implementation. Complete risk‐of‐bias evaluation results for all studies are presented in Figures S1–S3.

### 3.3 Pooled Meta‐Analyses and Sensitivity Analyses

For efficacy evaluation, we focused on two major categories of clinical outcomes: patient survival and lesion status. Analyses were conducted progressively, integrating both study design (controlled trials, single‐arm studies) and intervention type (monotherapy, combination therapy), with subgroup analyses stratified by cancer type, vaccine target, treatment line, EGFR status, and vaccine name.

First, we examined whether vaccine monotherapy, compared with best supportive care, improved patients' median OS and OS rate, including subgroups of NSCLC and GBM.

Second, we performed a proportional meta‐analysis of single‐arm studies evaluating vaccine monotherapy, with efficacy indicators including OS rate, ORR, and DCR. We explored differences in vaccine efficacy across treatment lines and whether such efficacy was primarily reflected in lesion shrinkage or control. These studies primarily included NSCLC patients.

Third, we further assessed whether combination therapy (vaccine added to standard regimens) was superior to standard treatment alone in terms of median OS, OS rate, median PFS, PFS rate, and ORR, encompassing both newly diagnosed and relapsed GBM patients. Sensitivity analyses explored the impact of residual lesion size on the efficacy of combination therapy.

Fourth, we conducted proportional analyses (stratified by cancer type, treatment line, and EGFR status) for efficacy indicators of vaccine combination regimens in single‐arm studies, including OS rate, PFS rate, ORR, and DCR. Sensitivity analyses investigated heterogeneity arising from differences in combination regimens and EGFR status.

Finally, for the safety analysis, we systematically summarized overall vaccine‐related TRAEs, the incidence of grade ≥ 3 TRAEs, and the specific types and their incidences of the most common TRAEs, with subgroup analyses stratified by vaccine name. Additionally, we comprehensively summarized common TRAEs in vaccine combination therapy.

#### 3.3.1 Pairwise Meta‐Analysis: Comparative Survival Benefits of EGF/EGFR Vaccines Versus Best Supportive Care

Three clinical studies evaluating EGF/EGFR‐targeted vaccine monotherapy versus best supportive care were included: 2 RCTs in NSCLC [33, 43] and 1 historical matched cohort study in GBM (ACTIVATE trial) [35].

**FIGURE 2:**
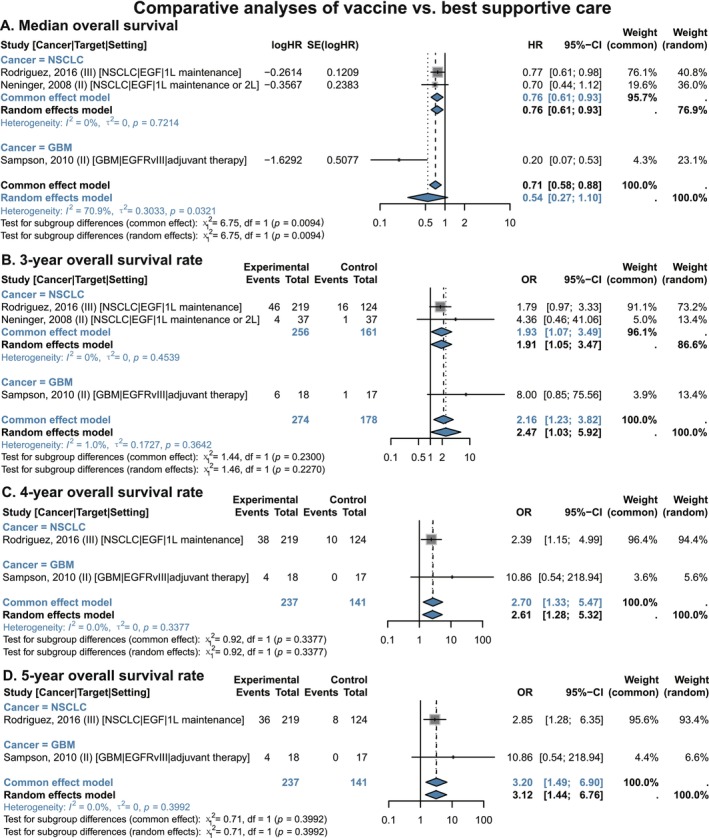
Comparative analyses of EGF/EGFR vaccines vs. best supportive care in NSCLC and GBM patients, stratified by cancer. (A) Median overall survival. (B) 3‐year Overall survival rate. (C) 4‐year Overall survival rate. (D) 5‐year Overall survival rate. 1 L, 1st‐line; 2 L, 2nd‐line; CI, confidence interval; EGF, epidermal growth factor; EGFRvIII, epidermal growth factor receptor variant III; GBM, glioblastoma; NSCLC, non‐small cell lung cancer; OR, odds ratio. Gray squares = individual study effect sizes (weighted by sample size). Blue diamonds = pooled effect sizes. Horizontal lines = 95% CI.

In the overall population, EGF/EGFR vaccine monotherapy significantly improved OS rates compared with best supportive care (Figure 2 and Figure S4). This population comprised two subgroups: NSCLC patients (who received EGF vaccine as first‐line maintenance or second‐line therapy) and GBM patients (who received EGFRvIII vaccine as adjuvant therapy). At different follow‐up time points, the pooled OS benefits for this population were as follows: 6‐month OS (OR 1.58, 95% CI 1.03–2.45), 1‐year OS (OR 1.68, 95% CI 1.12–2.52), 3‐year OS (OR 2.16, 95% CI 1.23–3.82), 4‐year OS (OR 2.70, 95% CI 1.33–5.47), and 5‐year OS (OR 3.20, 95% CI 1.49–6.90). All 95% CIs excluded 1, indicating significant survival benefits.

In the NSCLC subgroup, EGF‐targeted vaccine monotherapy significantly prolonged median OS (HR 0.76, 95% CI 0.61–0.93) and improved OS rates at 6 months (OR 1.56, 95% CI 1.01–2.42), 1 year (OR 1.58, 95% CI 1.04–2.38), and 3 years (OR 1.93, 95% CI 1.07–3.49) (Figure 2 and Figure S4).

Sensitivity analyses supported that EGF/EGFR vaccines confer long‐term survival benefits in NSCLC and GBM patients, thereby reinforcing the robustness of these findings (Figure S5).

#### 3.3.2 Single‐Arm Meta‐Analysis: EGF Vaccines Monotherapy Efficacy

We performed a pooled proportional analysis of efficacy indicators including OS rate, ORR, and DCR for vaccine monotherapy in single‐arm studies, with subgroup analyses stratified by treatment setting. A total of 7 NSCLC studies [36, 45, 46, 47, 48, 50] and 1 mixed cancer study (13/16 LC, 2/16 CRC, and 1/16 PC) [52] were included, all with EGF as the target.

**FIGURE 3:**
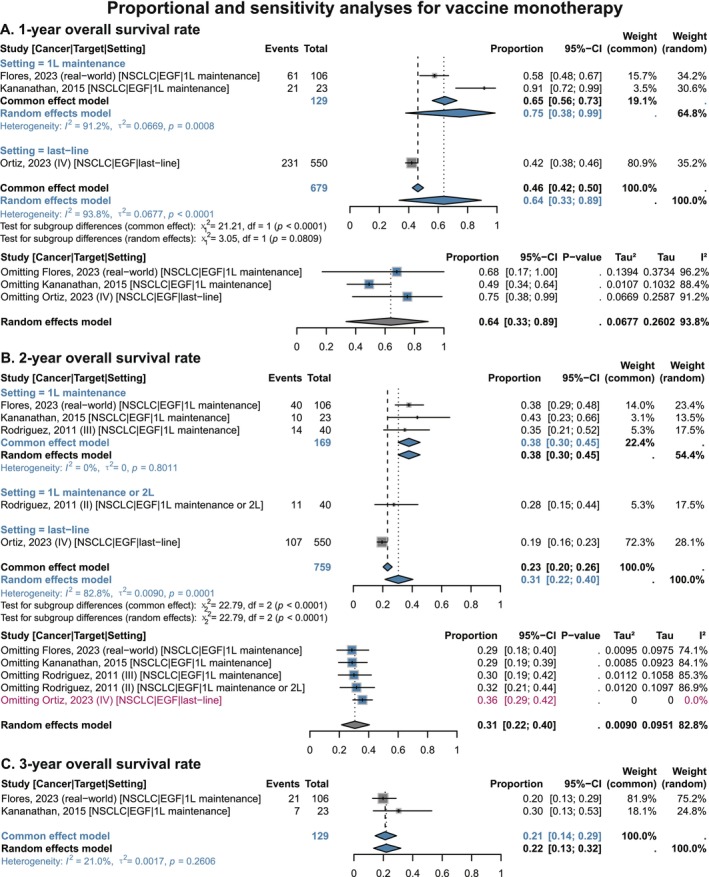
Proportional and sensitivity analyses for survival benefits of EGF vaccines in single‐arm NSCLC studies, stratified by treatment setting. (A) 1‐year Overall survival rate. (B) 2‐year Overall survival rate. (C) 3‐year Overall survival rate. NSCLC, non‐small cell lung cancer; EGF, epidermal growth factor; 1 L, 1st‐line; 2 L, 2nd‐line; CI, confidence interval. Gray squares = individual study effect sizes (weighted by sample size). Blue diamonds = pooled effect sizes. Horizontal lines = 95% CI. In sensitivity analysis, blue squares = effect sizes after excluding one individual study; gray diamonds = original overall pooled effect sizes.

In terms of OS benefits, NSCLC patients receiving vaccine monotherapy had an overall 1‐year rate of 64% (95% CI 0.33–0.89), 2‐year rate of 31% (95% CI 0.22–0.40), and 3‐year rate of 21% (95% CI 0.14–0.29) (Figure 3). In the subgroup receiving vaccine monotherapy as first‐line maintenance, OS estimates were higher: 1‐year rate of 75% (95% CI 0.38–0.99), 2‐year rate of 38% (95% CI 0.30–0.45), while the 3‐year value remained 21% (95% CI 0.14–0.29) (Figure 3). In contrast, OS rates were numerically lower in two subsets: patients receiving maintenance after first‐line chemotherapy or second‐line therapy (2‐year: 28% [95% CI 0.15–0.44]), and those receiving end‐line therapy (1‐year: 42% [95% CI 0.38–0.46]; 2‐year: 19% [95% CI 0.16–0.23]) (Figure 3). This suggests that earlier vaccine application may enhance long‐term survival benefits.

**FIGURE 4:**
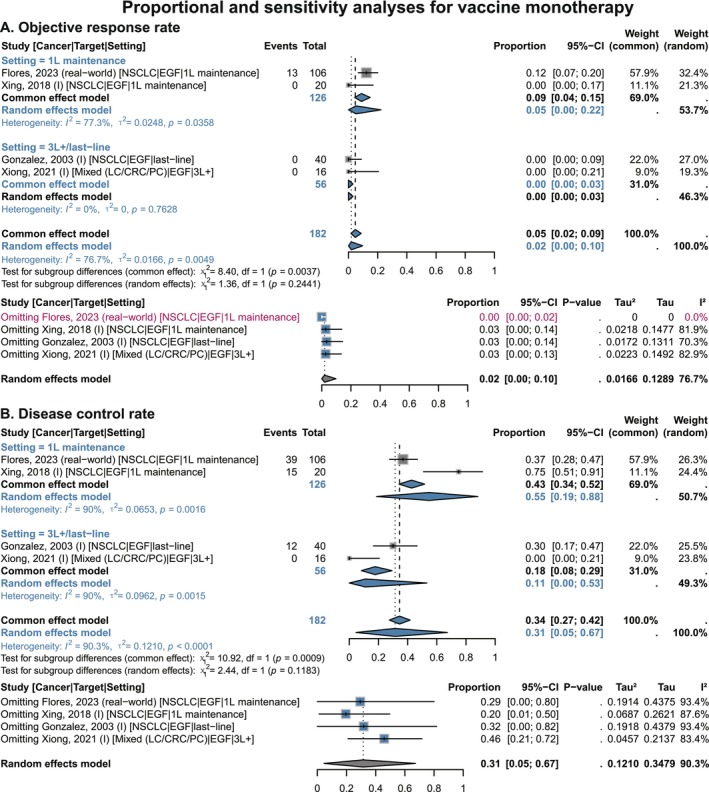
Proportional and sensitivity analyses of objective response rate and disease control rate benefits with EGF vaccines in single‐arm NSCLC and multi‐cancer studies, stratified by treatment setting. (A) Objective response rate. (B) Disease control rate. 1 L, 1st‐line; 2 L, 2nd‐line; 3 L+, ≥ 3rd‐line; CI, confidence interval; CRC: colorectal cancer; EGF, epidermal growth factor; LC, lung cancer; NSCLC, non‐small cell lung cancer; PC, pancreatic cancer. Gray squares = individual study effect sizes (weighted by sample size). Blue diamonds = pooled effect sizes. Horizontal lines = 95% CI. In sensitivity analysis, blue squares = effect sizes after excluding one individual study; gray diamonds = original overall pooled effect sizes.

We observed that the overall ORR and DCR of vaccine monotherapy were 2% (95% CI 0.00–0.10) and 31% (95% CI 0.05–0.67), respectively (Figure 4). When stratified by treatment lines, NSCLC patients receiving the vaccine as first‐line maintenance had a higher ORR (5%, 95% CI 0.00–0.22), whereas those with LC or other cancers receiving ≥ 3rd‐line treatment had an ORR of 0 (95% CI 0.00–0.03). However, the DCRs in these two subgroups were 55% (95% CI 0.19–0.88) and 11% (95% CI 0.00–0.53), respectively (Figure 4). This indicates that the efficacy of vaccine monotherapy is mainly reflected in lesion control rather than lesion regression, with better lesion control observed when applied earlier (Table 2).

**TABLE 2:** Pooled estimates and extracted data of EGF/EGFR vaccine therapies in NSCLC and GBM: survival and response outcomes.

Population	Outcomes (95% CI)							
	6mPFS	1yPFS	6mOS	1yOS	2yOS	3yOS	ORR	DCR
*EGF vaccine monotherapy*								
NSCLC [1Lmaintenance]	—	—	—	75% (0.38–0.99)	38% (0.30–0.45)	21% (0.14–0.29)	5% (0.00–0.22)	55% (0.19–0.88)
NSCLC [1Lmaintenance/2 L]	—	—	—	—	28% (0.15–0.44)	—	—	—
NSCLC [3 L/last‐line]	—	—	—	42% (0.38–0.46)	19% (0.16–0.23)	—	0% (0.00–0.03)	11% (0.00–0.53)
Overall	—	—	—	64% (0.33–0.89)	31% (0.22–0.40)	21% (0.14–0.29)	2% (0.00–0.10)	31% (0.05–0.67)
*EGF/EGFR vaccine combination therapy*								
NSCLC [1 L]	95% (0.76–1.00)	81% (0.58–0.95)	97% (0.81–1.00)	85% (0.54–1.00)	45% (0.03–0.92)	30% (0.04–0.65)	43% (0.11–0.79)	89% (0.80–0.95)
NSCLC [1 L maintenance/2 L]	72% (0.47–0.90)	33% (0.13–0.59)	89% (0.65–0.99)	67% (0.41–0.87)	50% (0.26–0.74)	—	33% (0.10–0.65)	50% (0.21–0.79)
TKI‐resistant [lines unknown]	—	—	—	—	—	—	45% (0.17–0.77)	100% (0.72–1.00)
GBM [adjuvant]	67% (0.55–0.77)	31% (0.13–0.52)	97% (0.91–1.00)	87% (0.77–0.94)	45% (0.34–0.57)	26% (0.16–0.39)	—	—
Overall	75% (0.57–0.90)	43% (0.18–0.70)	96% (0.92–0.99)	84% (0.77–0.90)	46% (0.30–0.62)	28% (0.14–0.46)	42% (0.21–0.64)	87% (0.70–0.98)

*Note:* Key statistical *p*‐values, including those for subgroup differences and heterogeneity analyses, can be referenced in the relevant forest plots (Figures 3, 4, 7, and 8; Figures S12 and S13).

Abbreviations: 1 L, 1st‐line; 1yOS, 1‐year overall survival; 1yPFS, 1‐year progression‐free survival; 2 L, 2nd‐line; 2yOS, 2‐year overall survival; 3 L, 3rd‐line; 3yOS, 3‐year overall survival; 6mOS, 6‐month overall survival; 6mPFS, 6‐month progression‐free survival; DCR, disease control rate; GBM, glioblastoma; NSCLC, non‐small cell lung cancer; ORR, objective response rate.

Sensitivity analyses indicated that pooled proportions for 1‐year OS, 2‐year OS, ORR, and DCR remained clinically stable after sequential exclusion of individual studies. For the 2‐year OS rate, exclusion of the phase IV study by Ortiz et al. (2023) (which enrolled end‐stage palliative patients) [36] completely eliminated heterogeneity while maintaining a stable pooled proportion of 36% (95% CI 0.29–0.42), indicating that patient population characteristics are a key source of heterogeneity (Figure 3). Heterogeneity in ORR was attributed to the NSCLC first‐line maintenance study by Flores et al. [45], which reported an ORR of 12%—in striking contrast to another first‐line maintenance study [48] and two studies involving therapy at ≥ 3rd‐lines [50, 52], all of which reported an ORR of 0% (Figure 4). Notably, the DCR in Flores et al. (2023) [45] (37%) was lower than that in Xing et al. (2018) [48] (75%). Therefore, heterogeneity in ORR may be related to differences in efficacy evaluation criteria and time windows.

Overall, these results indicate that EGF vaccine monotherapy confers survival benefits and lesion control effects in NSCLC, particularly in patients with relatively early‐stage disease.

#### 3.3.3 Pairwise Meta‐Analysis: Comparative Efficacy of EGFR Vaccine Combination Therapy Versus Standard Therapy

To examine whether adding EGF/EGFR vaccines to standard therapy could yield greater benefits, we further analyzed differences in patient survival and lesion response between vaccine‐containing combination regimens and standard regimens. A total of 3 GBM studies were included (2 for adjuvant therapy after maximal surgical resection and chemoradiation [39, 40], 1 for relapsed disease [34]), all utilizing the same EGFRvIII‐targeted vaccine. The control groups received TMZ (for adjuvant patients) [39, 40] or bevacizumab (for relapsed patients) [34].

**FIGURE 5:**
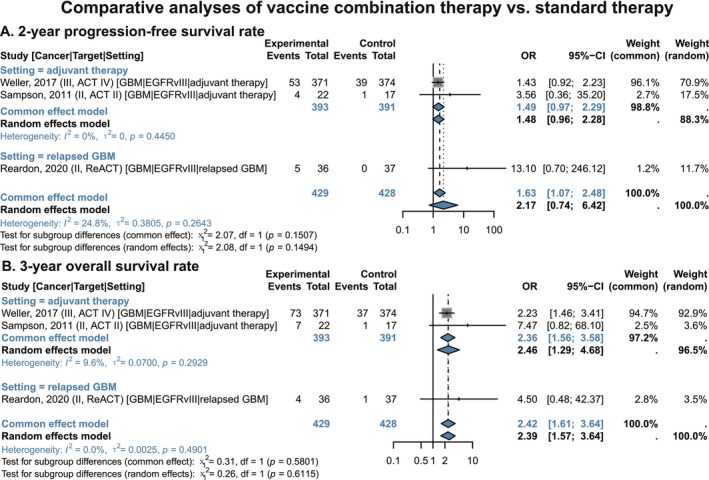
Comparative analyses of vaccine combination therapy vs. standard therapy in GBM patients, stratified by treatment setting. (A) 2‐year Progression‐free survival rate. (B) 3‐year Overall survival rate. CI, confidence interval; EGFRvIII, epidermal growth factor receptor variant III; GBM, glioblastoma; OR, odds ratio. Gray squares = individual study effect sizes (weighted by sample size). Blue diamonds = pooled effect sizes. Horizontal lines = 95% CI.

Pooled results showed that in the overall population of newly diagnosed and relapsed GBM, the vaccine combination group had a higher 3‐year OS rate (OR 2.42, 95% CI 1.61–3.64) and 2‐year PFS rate (OR 1.63, 95% CI 1.07–2.48) than the standard therapy alone group (Figure 5). In the adjuvant therapy subgroup, adding vaccine to TMZ significantly improved the 3‐year OS rate (OR 2.36, 95% CI 1.56–3.58), with a trend toward improved 2‐year PFS rate (OR 1.49, 95% CI 0.97–2.29) (Figure 5). No significant differences were observed in median survival, short‐term survival rates, or ORR (Figures S6 and S7).

Sensitivity analyses identified the non‐randomized historical control study by Sampson et al. (2011) [39] as the main source of heterogeneity in PFS benefits (Figure S8). More importantly, after excluding data from the intent‐to‐treat (ITT) population of the “ACT IV” study, the combination therapy group showed improvements in median OS (HR 0.42, 95% CI 0.20–0.87), 2‐year OS rate (OR 10.73, 95% CI 2.31–49.73), and 1‐year PFS rate (OR 3.26, 95% CI 1.17–9.11) in newly diagnosed and relapsed GBM patients, compared with standard therapy alone (Figures S8 and S9).

**FIGURE 6:**
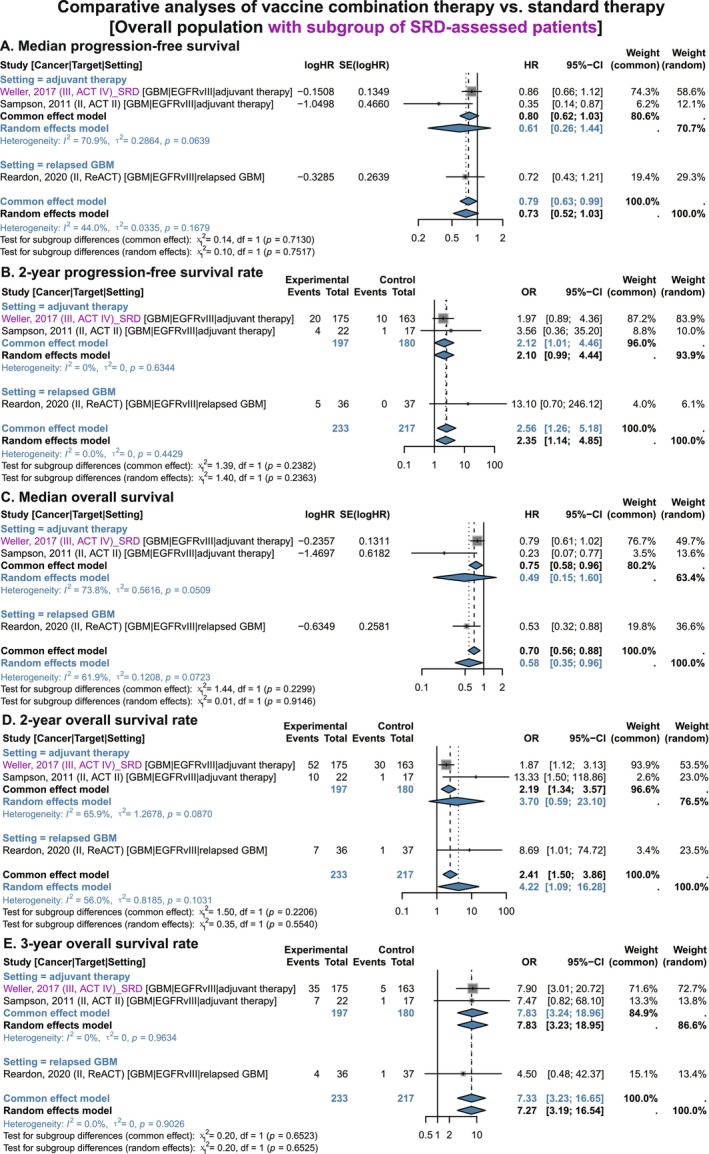
Comparative analyses of vaccine combination therapy vs. standard therapy in GBM patients (overall population with subgroup of SRD‐assessed patients), stratified by treatment setting. (A) Median progression‐free survival. (B) 2‐year Progression‐free survival rate. (C) Median overall survival. (D) 2‐year Overall survival rate. (E) 3‐year Overall survival rate. CI, confidence interval; EGFRvIII, epidermal growth factor receptor variant III; GBM, glioblastoma; HR, hazard ratio; OR, odds ratio; SRD, significant residual disease. Gray squares = individual study effect sizes (weighted by sample size). Blue diamonds = pooled effect sizes. Horizontal lines = 95% CI.

The “ACT IV” study classified enrolled patients into subgroups with significant residual disease (SRD, ≥ 2 cm^2^) and minimal residual disease (MRD, < 2 cm^2^) based on residual lesion size [40]. Therefore, we pooled data from SRD and MRD populations with those from the other two studies, respectively. Results showed that when including the SRD population from the “ACT IV” study, GBM patients receiving vaccine combination therapy had larger effect sizes for 3‐year OS rate (OR 7.33, 95% CI 3.23–16.65) and 2‐year PFS rate (OR 2.56, 95% CI 1.26–5.18) compared with the overall population mentioned above. More importantly, combination therapy was significantly superior to standard therapy alone in terms of median OS (HR 0.58, 95% CI 0.35–0.96), 2‐year OS rate (OR 4.22, 95% CI 1.09–16.28), and median PFS (HR 0.79, 95% CI 0.63–0.99) (Figure 6). In the adjuvant therapy subgroup, combination therapy showed a more significant improvement in 3‐year OS rate (OR 7.83, 95% CI 3.24–18.96), with the improvement in 2‐year PFS rate also reaching statistical significance (OR 2.12, 95% CI 1.01–4.46) (Figure 6). However, after pooling MRD patient data from the “ACT IV” study with the other two studies, consistent non‐significant results were observed across all indicators (Figures S10 and S11).

These findings indicate that EGFRvIII vaccine combined with standard therapy, compared with standard therapy alone, may prolong OS and PFS in patients with GBM, with a trend toward more pronounced survival benefits in those with SRD.

#### 3.3.4 Single‐Arm Meta‐Analysis: EGF/EGFR Vaccines Combination Regimens

We further performed a pooled proportional analysis of efficacy indicators from single‐arm studies exploring EGF/EGFR vaccine‐containing combination regimens, with subgroup stratification by cancer type, treatment line, and EGFR status. A total of 6 NSCLC studies (5 targeting EGF [38, 54, 56, 57, 58], 1 targeting EGFR [37]) and 2 GBM adjuvant therapy studies (both targeting EGFRvIII) [44, 59] were included in the pooled analysis. Results showed that vaccine combination therapy exhibited clear therapeutic effects in terms of time‐point survival rates, lesion response, and disease control (Table 2).

**FIGURE 7:**
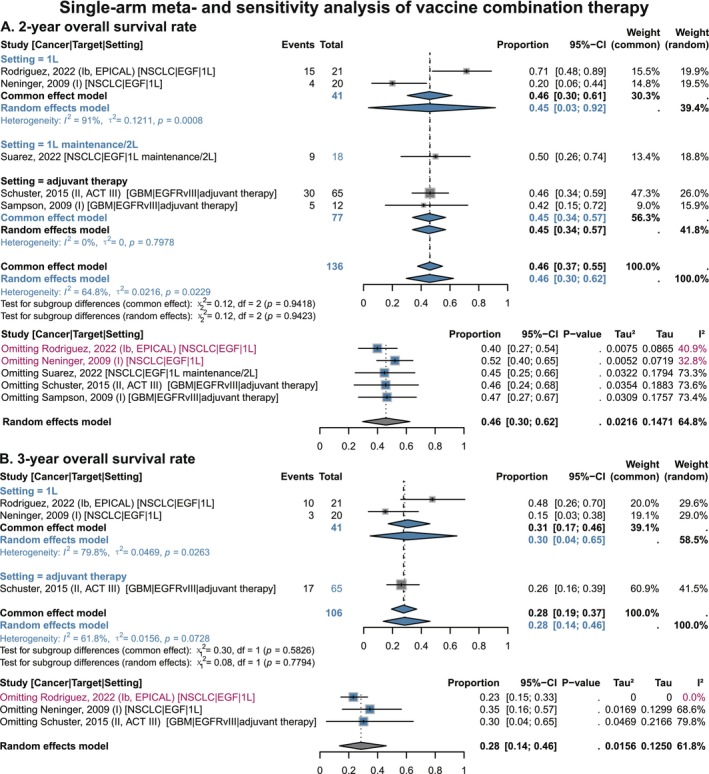
Proportional and sensitivity analyses of overall survival benefits of EGF/EGFR vaccines combination therapy in single‐arm NSCLC and GBM studies, stratified by treatment setting. (A) 2‐year Overall survival rate. (B) 3‐year Overall survival rate. 1 L, 1st‐line; 2 L, 2nd‐line; CI, confidence interval; EGF, epidermal growth factor; EGFRvIII, epidermal growth factor receptor variant III; GBM, glioblastoma; NSCLC, non‐small cell lung cancer. Gray squares = individual study effect sizes (weighted by sample size). Blue diamonds = pooled effect sizes. Horizontal lines = 95% CI. In sensitivity analysis, blue squares = effect sizes after excluding one individual study; gray diamonds = original overall pooled effect sizes.

**FIGURE 8:**
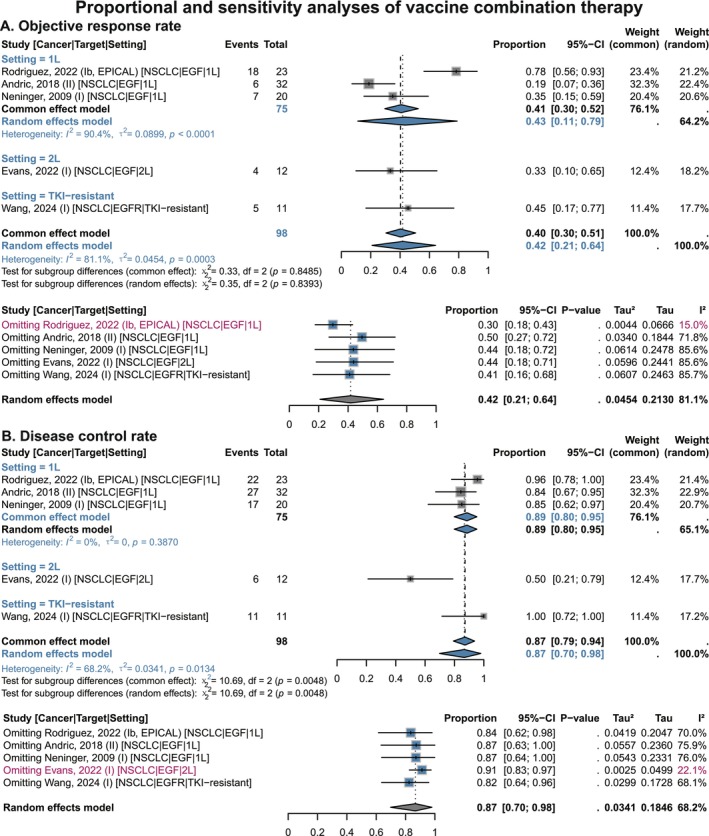
Proportional and sensitivity analyses of objective response rate and disease control rate benefits of EGF/EGFR vaccines combination therapy in single‐arm NSCLC studies, stratified by treatment setting. (A) Objective response rate. (B) Disease control rate. 1 L, 1st‐line; 2 L, 2nd‐line; CI, confidence interval; EGF, epidermal growth factor; EGFR, epidermal growth factor receptor; NSCLC, non‐small cell lung cancer; TKI, tyrosine kinase inhibitor. Gray squares = individual study effect sizes (weighted by sample size). Blue diamonds = pooled effect sizes. Horizontal lines = 95% CI. In sensitivity analysis, blue squares = effect sizes after excluding one individual study; gray diamonds = original overall pooled effect sizes.

Overall, vaccine combination therapy showed favorable survival benefits in single‐arm analyses (Figure S12 and Figure 7). Specifically, the 6‐month OS rate was 96% (95% CI 0.92–0.99), the 1‐year OS rate was 84% (95% CI 0.77–0.90), the 2‐year OS rate was 46% (95% CI 0.30–0.62), and the 3‐year OS rate was 28% (95% CI 0.14–0.46). In terms of PFS, the combination therapy also yielded positive results (Figure S13): the 6‐month PFS rate was 75% (95% CI 0.57–0.90) and the 1‐year PFS rate was 43% (95% CI 0.18–0.70). For lesion response indicators (Figure 8), the pooled ORR was 42% (95% CI 0.21–0.64) and DCR was 87% (95% CI 0.70–0.98), further confirming the therapeutic effect.

In the NSCLC subgroup, first‐line patients had the following survival outcomes: 6‐month OS rate of 97% (95% CI 0.81–1.00), 1‐year OS rate of 85% (95% CI 0.54–1.00), 2‐year OS rate of 45% (95% CI 0.03–0.92), and 3‐year OS rate of 30% (95% CI 0.04–0.65) (Figure S12 and Figure 7). For lesion response, these patients had an ORR of 43% (95% CI 0.11–0.79) and a DCR of 89% (95% CI 0.80–0.95) (Figure 8).

In the GBM adjuvant therapy subgroup, 6‐month, 1‐year, 2‐year, and 3‐year OS rates were 97% (95% CI 0.91–1.00), 87% (95% CI 0.77–0.94), 45% (95% CI 0.34–0.57), and 26% (95% CI 0.16–0.39), respectively (Figure S12 and Figure 7); and 6‐month and 1‐year PFS rates were 67% (95% CI 0.55–0.77) and 31% (95% CI 0.13–0.52), respectively (Figure S13).

Subgroup analysis by EGFR status showed that the pooled ORR and DCR of EGFR‐mutant NSCLC patients receiving vaccine combination therapy were 65% (95% CI 0.31–0.92) and 98% (95% CI 0.88–1.00), respectively. These values were the most prominent among all patients and higher than those in patients with EGFR wild‐type or unknown EGFR status (Figure S14).

Sensitivity analysis revealed that vaccine combination therapy showed consistent directional and clinical stability for OS, PFS, ORR, and DCR benefits (Figures 7 and 8; Figures S12 and S13). The EPICAL study [54] was the main source of heterogeneity across multiple indicators. This study combined EGF vaccine with the second‐generation EGFR‐TKI afatinib in first‐line EGFR‐mutant NSCLC patients, and its reported OS rate, PFS rate, ORR, and DCR were the most prominent among all included studies in the pooled analysis. The study by Neninger et al. (2009), which explored EGF vaccine combined with chemotherapy in first‐line NSCLC [57], was the main source of heterogeneity in 2‐year and 3‐year OS rates, with relatively lower efficacy. Heterogeneity in DCR was primarily attributed to the study by Evans et al. (2022) [38]. This study enrolled second‐line NSCLC patients treated with the EGF vaccine combined with the ICI nivolumab, yielding a DCR of only 50%—in contrast to the other four studies, which all reported a DCR of over 84% [37, 54, 56, 57].

These findings suggest that the efficacy of vaccine combination regimens is highly dependent on patient baseline characteristics, types of combined drugs, and treatment lines. Future efforts should focus on optimizing combination strategies for preferential populations to avoid the risk of low response.

#### 3.3.5 Safety Profile of EGF/EGFR Vaccines: Monotherapy and Combination Therapy

We analyzed the incidence of overall, grade ≥ 3, and the most common TRAEs in patients receiving vaccine monotherapy. For the meta‐analysis, the included studies comprised two RCTs comparing EGF vaccine monotherapy with best supportive care (with data extracted exclusively from the monotherapy group) [43, 49] and seven single‐arm studies [33, 36, 45, 48, 50, 51, 52].

Overall, the vaccines exhibited good safety and tolerability. TRAEs induced by vaccine monotherapy were mostly mild (grade 1) to moderate (grade 2), primarily including injection site reactions, chills, fever, headache, nausea, and vomiting. Grade ≥ 3 TRAEs were infrequently observed (Table 1), and no vaccine‐related serious adverse events (SAEs) or dose‐limiting toxicities (DLTs) were reported.

For the calculation of TRAEs incidence, we divided subgroups according to vaccine names. In the vaccine monotherapy cohort, the cumulative incidence of overall TRAEs was 62% (95% CI 0.53–0.70) (Figure S15). Among local reactions, the incidence point estimates for pain, induration, and erythema were 29% (95% CI 0.16–0.44), 19% (95% CI 0.00–0.55), and 9% (95% CI 0.04–0.15), respectively (Figure S16). For systemic events, the incidence rates of chills and fever were 35% (95% CI 0.15–0.58) and 23% (95% CI 0.10–0.38), respectively (Figure S17). The incidence of headache (a neurological event) was 22% (95% CI 0.18–0.27) (Figure S18), while the occurrence rates of nausea and vomiting (gastrointestinal events) were 18% (95% CI 0.06–0.33) and 19% (95% CI 0.06–0.37), respectively (Figure S19). In the subgroup of CIMAvax‐EGF, the overall incidence of TRAEs was 59% (95% CI 0.45–0.71), with grade ≥ 3 TRAEs occurring in 2% (95% CI 0.00–0.06) of cases (Figure S15). The estimated incidence of injection site pain was 36% (95% CI 0.22–0.52) (Figure S16); chills and fever had incidence rates of 35% (95% CI 0.15–0.58) and 24% (95% CI 0.09–0.44), respectively (Figure S17); headache occurred in 23% (95% CI 0.18–0.28) of subjects (Figure S18); and the frequencies of nausea and vomiting were 21% (95% CI 0.07–0.39) and 19% (95% CI 0.06–0.37), respectively (Figure S19). Sensitivity analyses confirmed the robustness of results following exclusion of individual studies.

For combination therapy, formal AEs comparison between vaccine combination therapy and standard therapy was not performed due to limited comparative data. Among the three controlled studies available, one reported TRAEs [34], one reported all‐cause AEs [40], and another (with historical control) lacked control group AE data [39], precluding direct comparison. Notably, available data suggested AE incidence in the combination group was comparable to standard therapy alone for both TRAEs [34], and all‐cause AEs [40]. To aid reference on combination therapy safety, we summarized its safety profile from relevant arms of controlled and single‐arm studies (Table S4). These studies showed no new AE categories, with all SAEs attributed to concomitant medications (e.g., ICIs) [38, 51].

## 4 Discussion

This systematic review and meta‐analysis integrated data from 26 clinical trials to comprehensively evaluate EGF/EGFR vaccines in EGFR‐driven solid tumors. We observed both monotherapy and combination therapy conferred significant survival benefits in these tumors (particularly NSCLC and GBM) with favorable safety profiles. Compared with best supportive care, monotherapy notably improved long‐term OS in NSCLC and GBM. In first‐line maintenance therapy for NSCLC, the 1‐year OS rate reached 75%. Disease control may be the primary therapeutic strength of vaccine monotherapy. Vaccine combination therapy further showed promising efficacy trends. In GBM, combination regimens improved OS and PFS, with this advantage being particularly prominent when the subgroup with SRD was included in the pooled analysis (3‐year OS rate: OR = 7.33). In NSCLC, vaccine combination therapy appeared to outperform monotherapy in ORR and DCR, with the 1‐year OS rate reaching 85% in first‐line settings; notably, patients with EGFR mutations derived more prominent benefits, with a pooled ORR of 65% and a DCR of 98%, though these findings require validation in larger cohorts. Regarding safety, TRAEs associated with the vaccines were mainly grade 1–2, with no vaccine‐related SAEs or DLTs reported, demonstrating good tolerability; preliminary data also suggested that vaccine combination therapy did not induce additional AEs. These findings indicate that EGF/EGFR vaccines possess both targeting ability and immunogenicity and hold potential for clinical translation in specific EGFR‐driven solid tumors (e.g., NSCLC and GBM). However, their efficacy appears to be closely linked to treatment timing, tumor burden, and combination regimens and cannot be broadly generalized to all EGFR‐driven solid tumors—evidence in other EGFR‐overexpressing malignancies (e.g., prostate cancer, CRC) remains limited, and further validation is needed.

EGF/EGFR vaccine monotherapy exhibits “low ORR but significant survival benefits”, which contrasts with traditional cytotoxic therapies that “focus on tumor shrinkage”. Their therapeutic essence lies in “delaying disease progression” rather than “rapidly reducing tumors”, as evidenced by low ORR (2%) and favorable DCR (31%). Our results showed that compared with best supportive care, vaccine monotherapy increased the 3‐year OS rate by 2.16‐fold and the 5‐year OS rate by 3.20‐fold in patients with NSCLC and GBM, confirming a significant long‐term survival advantage of monotherapy. The survival benefit stems from the vaccine's dual mechanisms. First, vaccine‐induced anti‐EGF antibodies neutralize circulating EGF, directly inhibiting EGFR signaling pathway activation and suppressing tumor growth [33, 64, 65]. Even though three single‐arm studies reported an ORR of 0%, all patients had elevated, sustained serum anti‐EGF antibody titers [48, 50, 52]. These titers were negatively correlated with circulating EGF levels [48], and the magnitude of the antibody response correlated with OS and PFS [52]. Second, the vaccine induces tumor antigen‐specific T cell infiltration into the tumor microenvironment; memory T cells then clear micrometastases via long‐term immune surveillance, reducing recurrence risk [33, 54, 59, 66, 67, 68, 69, 70, 71]. Notably, NSCLC patients receiving vaccine monotherapy as first‐line maintenance therapy had a higher OS rate than those receiving later‐line therapy, suggesting that the long‐term survival benefits conferred by the vaccine are more pronounced in patients with early intervention.

As an immunotherapy, traditional efficacy evaluation criteria may lead to biased assessments of the actual efficacy of vaccines [72, 73]. In NSCLC vaccine monotherapy studies, Flores et al. (2023) used Response Evaluation Criteria in Solid Tumors (RECIST) 1.1 for assessments at 6 months and 1 year (ORR = 12%, DCR = 37%) [45]. In contrast, Xing et al. (2018) used the same criteria but with earlier assessments (46 days ±1 week; ORR = 0%, DCR = 75%) [48]. Among 3rd‐line and beyond patients, Xiong et al. (2021) used early RECIST 1.1 assessments (every 8 weeks; DCR = 0%) [52], while Gonzalez et al. (2003) used WHO criteria (assessments every 3 months after the first month) and reported a DCR of 30% [50]. This suggests that differences in efficacy evaluation criteria and assessment time windows may influence the results. Due to the unique characteristics of vaccines—‘'delayed response’' and “pseudoprogression”—early assessment tends to underestimate DCR because immune‐mediated delayed tumor shrinkage has not yet occurred, while late assessment may overestimate efficacy due to natural tumor fluctuations. Thus, the immune‐related RECIST (irRECIST), which considers the kinetics of immune responses, may be more suitable for evaluating vaccine efficacy [74, 75, 76]. In future vaccine studies, efficacy evaluations can compare irRECIST and RECIST criteria, coupled with immune biomarkers, rather than relying solely on traditional imaging criteria.

Combination therapy further amplifies the clinical value of EGF/EGFR vaccines, with consistent benefits observed across different EGFR‐driven tumors. In our analysis, for GBM, EGFRvIII vaccine combined with standard therapy (TMZ/bevacizumab) resulted in significantly higher 3‐year OS rate (OR = 2.42) and 2‐year PFS rate (OR = 1.63) compared with standard therapy alone. Mechanistically, vaccine administration induces a significant increase in antibody titers, and high antibody titers correlate with longer OS [34, 39, 40], providing an immunological basis for the synergistic effect of combination therapy. The tumor‐reducing effect of TMZ and the immune activation induced by the vaccine form a synergistic “antigen release‐immune clearance” cycle [77, 78]; furthermore, TMZ‐induced lymphopenia upregulates serum B lymphocyte stimulator (BLyS) levels, and BLyS can enhance antigen‐specific antibody titers [79]. EGFRvIII vaccine combined with bevacizumab can synergistically enhance anti‐tumor effects through bidirectional “immune activation‐vascular regulation” interactions [80, 81]. Notably, we observed no significant prolongation in median OS or median PFS in the EGFRvIII vaccine combination therapy group, which may also be related to the “delayed effect.” Vaccine‐induced antigen‐specific memory T cells require long‐term accumulation to gradually manifest survival benefits. This effect is difficult to capture by short‐term median OS, while long‐term survival rates better reflect its durable benefits [82, 83, 84, 85, 86].

In the “ACT IV” trial, the bidirectional dilution effect between the SRD and MRD subgroups is central to understanding the results of GBM combination therapy [40]. In the “ACT IV” study, the combination therapy group included 47% of patients with SRD and 53% with MRD; the TMZ group included 44% of patients with SRD and 56% with MRD. In the SRD subgroup, vaccine combined with TMZ resulted in a 2‐year OS rate of 30% (vs. 19% in the TMZ monotherapy group), with separation of survival curves (HR = 0.79); however, in the MRD subgroup, no significant difference was observed between combination therapy and monotherapy (HR = 1.01). When data from both groups were pooled, the dominant proportion of MRD patients weakened the overall effect, resulting in a pooled HR of 0.58 (95% CI 0.31–1.10) for the total ITT population. After pooling data from the SRD subgroup with all cases from the other two unstratified studies, we found that the benefits in this pooled population were particularly prominent: the 3‐year OS rate had an OR of 7.33, with both median OS (HR = 0.58) and median PFS (HR = 0.79) significantly prolonged. This may be related to the complexity of immune responses. In the SRD population, sustained stimulation from abundant tumor antigens may activate a more comprehensive immune response network (e.g., infiltration of antigen‐specific T cells, increased cytokine secretion) [87]. However, in the MRD population, even with sufficient antibody levels, a mere antibody response cannot be translated into survival benefits if residual tumor lesions fail to provide sufficient antigens to sustain immune activation or if residual cells evade humoral immune attack through immune escape mechanisms [87]. This finding emphasizes that (1) tumor burden is a key regulator of vaccine efficacy; (2) future trials may consider stratification by residual lesion size; (3) for the MRD population, strategies such as “vaccine + ICIs” or “vaccine + anti‐angiogenic drugs” need to be explored to overcome antigen insufficiency and microenvironmental suppression.

Through pooled analysis of single‐arm studies, we observed that combination therapy also confers clear benefits to NSCLC patients. As shown in Table 2, a horizontal comparison suggests that vaccine combination regimens outperform monotherapy in ORR and DCR. In multiple studies of vaccine combination therapy for NSCLC, vaccinated patients exhibited significantly elevated antigen‐specific antibody titers [54, 55, 57, 58], which were strongly negatively correlated with serum EGF levels [38, 58]; additionally, “high‐titer responders” had significantly better OS than low‐titer responders [57]. More importantly, in terms of cellular immune response, the vaccine can induce specific T‐cell responses against EGFR neoantigen peptides [37, 51], accompanied by upregulated serum Th1‐type cytokines and a marked increase in the proportion of effector memory T cells in peripheral blood [37]. This mechanistically explains the synergistic advantages of combining vaccines with other anti‐tumor approaches. Notably, we further observed that first‐line patients achieved better survival benefits and lesion response/control than those on first‐line maintenance or second‐line therapy, which further suggests that early application of vaccine combination therapy may improve patient prognosis. Sensitivity analysis identified the 2022 study by Evans et al. as the main source of DCR heterogeneity in vaccine combination therapy [38]. This study included second‐line NSCLC patients treated with an EGF vaccine combined with ICI (nivolumab), achieving a DCR of only 50%; in contrast, the other four studies (first‐line: EGF vaccine plus TKI or chemotherapy [54, 56, 57]; TKI‐resistant: EGF vaccine plus ICI and chemotherapy [37]) all had a DCR exceeding 84%. This finding suggests that in clinical practice, treatment timing and combination regimens should be comprehensively considered based on patient characteristics to optimize the efficacy of vaccine‐based combination therapy.

A study by Wang et al. showed that even with TKI resistance, nearly half of NSCLC patients with EGFR mutations achieved lesion remission after receiving a vaccine combined with tislelizumab and chemotherapy, with a DCR of 100% [37]. In the EPICAL study, first‐line EGFR‐mutant patients receiving a vaccine combined with afatinib had a median OS of 26.9 months, a median PFS of 17.5 months, an ORR of 78.3%, and a DCR of 95.7% [54]. In contrast, first‐line NSCLC patients with EGFR wild‐type tumors [56] or unknown EGFR status [55, 57] derive limited benefits from the EGF vaccine combined with chemotherapy. Our subgroup analysis of ORR and DCR stratified by EGFR status confirms that NSCLC patients with EGFR mutations are the dominant beneficiaries of vaccine combination therapy. This advantage may be attributed to the mechanism by which EGFR mutations can increase tumor antigens, enhance vaccine‐induced T cell recognition, and thereby promote the anti‐tumor immune response of the vaccine [26, 28, 54]. Therefore, EGF/EGFR vaccine combination therapy is a highly promising preferred immunotherapeutic strategy for EGFR‐mutant tumors, especially in scenarios where ICIs have limited efficacy [88, 89].

Beyond GBM and NSCLC, the benefits of vaccine combination therapy have also been preliminarily validated in other EGFR‐driven tumors. In castration‐resistant prostate cancer, one RCT—excluded from the meta‐analysis due to data limitations—showed that the median OS of patients in the EGF vaccine plus chemotherapy group reached 17.1 months (vs. 10.73 months in the chemotherapy‐alone group), with 2‐year and 3‐year OS rates increased by 9.15% and 8.84%, respectively [53]. Mechanistically, in patients receiving the EGFR vaccine, EGFR‐specific IFN‐γ + T‐cell responses were significantly associated with survival [90]. Thus, the benefits of vaccine therapy in castration‐resistant prostate cancer are consistent with those observed in NSCLC and GBM, collectively supporting the conclusion that “EGF/EGFR vaccine‐based combination therapy can prolong survival in patients with EGFR‐driven malignancies” and highlighting its potential for cross‐tumor application.

The safety profile of EGF/EGFR vaccines is a key advantage that distinguishes them from other immunotherapeutic modalities. Most TRAEs are grade 1–2, with common manifestations including injection site reactions, fever, headache, nausea, and vomiting. The incidence of grade ≥ 3 TRAEs is extremely low, with no vaccine‐related SAEs or DLTs reported. This safety profile contrasts sharply with that of ICIs. ICIs frequently induce multisystem immune‐related AEs (e.g., colitis, pneumonia) [91, 92, 93], whereas the high target specificity of vaccines enables selective activation of EGF/EGFR‐specific immune responses, thereby reducing the risk of systemic autoimmune activation [66, 67, 94, 95]. This low toxicity and targeted specificity make them a promising option for maintenance therapy, especially in vulnerable patients (e.g., those with poor performance status or comorbid underlying conditions), or individuals with limited treatment options after multiple lines of therapy. For these later‐line patients, although vaccine efficacy may be inferior to that observed in first‐line settings, the low toxicity profile still reduces the risk of treatment discontinuation and enables sustained disease control.

This study has several limitations. First, as an observational study based on existing clinical data, its conclusions require validation via prospective investigations. Additionally, inherent constraints in the data and design of included studies introduce limitations: (1) insufficient studies for publication bias assessment; (2) small sample sizes compromise result stability; (3) limited long‐term survival data impede comprehensive evaluation of long‐term efficacy. Furthermore, evidence on EGFR‐overexpressing tumors (e.g., prostate cancer, CRC) remains sparse, restricting conclusion extrapolation. Finally, few included RCTs (2/5 with high bias risk), 2 non‐randomized controlled studies with weak evidential strength, and most evidence from single‐arm studies (which may overestimate efficacy) further limit interpretation.

Future research could address several key directions. Notably, conducting large‐scale multicenter RCTs is critical to verifying long‐term benefits, while multi‐omics approaches should be employed to clarify the predictive value of EGFR status and immune biomarkers [96, 97, 98, 99, 100]. Meanwhile, extending research to EGFR‐overexpressing tumors will help fill current evidence gaps, such as prostate cancer, CRC, and head and neck squamous cell carcinoma (HNSCC). Standardizing efficacy evaluation criteria and optimizing combination regimens for populations with MRD are also imperative. Additionally, enhancing immunogenicity through the integration of mRNA vaccines and nano‐delivery systems represents a promising strategy to improve vaccine performance [101, 102, 103, 104, 105]. Several clinical trials are ongoing to accumulate evidence for EGF/EGFR vaccines. For instance, a phase I/II trial is investigating CIMAvax‐EGF combined with ICIs in NSCLC and HNSCC [106], a phase 0 study is accumulating data in CRC [107], and another is recruiting to explore the potential of CIMAvax‐EGF for preventing LC recurrence and reducing incidence in high‐risk groups [108]. These studies will further facilitate the clinical translation of EGF/EGFR vaccines.

In summary, through the dual mechanisms of targeted EGFR pathway inhibition and antigen‐specific immunity activation, EGF/EGFR vaccines have shown potential survival benefits in EGFR‐driven solid tumors, along with favorable safety profiles supported by current data. Monotherapy may be a promising option for early maintenance to delay disease progression, while combination therapy has exhibited initial trends of amplified efficacy in NSCLC, GBM, and prostate cancer—with more notable responses observed in specific subgroups (e.g., patients with SRD or EGFR mutations). However, these benefits and the vaccines' broader clinical value require validation in larger, high‐quality cohorts. Their unique efficacy pattern (prioritizing disease control over rapid tumor shrinkage) and low toxicity suggest they could serve as a valuable complementary option to existing treatments for selected patients. Looking ahead, optimizing the efficacy evaluation system (e.g., validating irRECIST applicability), clarifying predictive biomarkers (e.g., EGFR status, immune response indicators), and expanding research to more EGFR‐driven tumor types may enable EGF/EGFR vaccines to play a role in precision immuno‐oncology.

## Author Contributions

**Fei Chen:** investigation, writing – original draft, methodology, validation, writing – review and editing, software, formal analysis, data curation, visualization. **Ling Bai:** conceptualization, investigation, funding acquisition, writing – review and editing, visualization, validation, data curation, methodology, writing – original draft. **Jiuwei Cui:** conceptualization, funding acquisition, writing – review and editing, supervision, investigation, project administration, writing – original draft.

## Ethics Statement

The authors have nothing to report.

## Conflicts of Interest

The authors declare no conflicts of interest.

## Supporting information


**Table S1:** PRISMA 2020 Checklist.
**Table S2:** Search strategies of possible publications.
**Table S3:** Time‐specific survival rates and calculated HR data digitized from survival curves.
**Table S4:** The most common therapy‐related AEs in vaccine combination therapy.
**Figure S1:** Risk of bias assessment for the included non‐randomized controlled trials and single‐arm studies, according to the MINORS Evaluation Criteria.
**Figure S2:** Risk of bias assessment of randomized controlled trials assessing EGF/EGFR vaccines vs. best supportive care. (A) Risk of bias summary (judgments about each risk of bias item for each included study). (B) Risk of bias graph (judgments about each risk of bias item presented as percentages across all included studies).
**Figure S3:** Risk of bias assessment of randomized controlled trials assessing EGF/EGFR vaccines combination therapy vs. controlled onco‐specific treatment alone. (A) Risk of bias summary (judgments about each risk of bias item for each included study). (B) Risk of bias graph (judgments about each risk of bias item presented as percentages across all included studies).
**Figure S4:** Comparative analyses of EGF/EGFR vaccines vs. best supportive care in NSCLC and GBM patients, stratified by cancer. (A) 6‐month Overall survival rate. (B) 1‐year Overall survival rate. (C) 2‐year Overall survival rate. 1 L, 1st‐line; 2 L, 2nd‐line; CI, confidence interval; EGF, epidermal growth factor; EGFRvIII, epidermal growth factor receptor variant III; GBM, glioblastoma; NSCLC, non‐small cell lung cancer; OR, odds ratio. Gray squares = individual study effect sizes (weighted by sample size). Blue diamonds = pooled effect sizes. Horizontal lines = 95% CI.
**Figure S5:** Sensitivity analyses for pooled benefits of EGF/EGFR vaccines vs. best supportive care in NSCLC and GBM patients. (A) Median overall survival. (B) 6‐month Overall survival rate. (C) 1‐year Overall survival rate. (D) 2‐year Overall survival rate. (E) 3‐year Overall survival rate. 1 L, 1st‐line; 2 L, 2nd‐line; CI, confidence interval; EGF, epidermal growth factor; EGFRvIII, epidermal growth factor receptor variant III; GBM, glioblastoma; NSCLC, non‐small cell lung cancer; OR, odds ratio. Blue squares = effect sizes after excluding one individual study. Gray diamonds = original overall pooled effect sizes.
**Figure S6:** Comparative analyses of vaccine combination therapy vs. standard therapy in GBM patients, stratified by treatment setting. (A) Median progression‐free survival. (B) 6‐month Progression‐free survival rate. (C) 1‐year Progression‐free survival rate. (D) Objective response rate. CI, confidence interval; GBM, glioblastoma; HR, hazard ratio; OR, odds ratio. Gray squares = individual study effect sizes (weighted by sample size). Blue diamonds = pooled effect sizes. Horizontal lines = 95% CI.
**Figure S7:** Comparative analyses of vaccine combination therapy vs. standard therapy in GBM patients, stratified by treatment setting. (A) Median overall survival. (B) 6‐month Overall survival rate. (C) 1‐year Overall survival rate. (D) 2‐year Overall survival rate. CI, confidence interval; GBM, glioblastoma; HR, hazard ratio; OR, odds ratio. Gray squares = individual study effect sizes (weighted by sample size). Blue diamonds = pooled effect sizes. Horizontal lines = 95% CI.
**Figure S8:** Sensitivity analyses for progression‐free survival benefits of vaccine combination therapy vs. standard therapy in GBM patients. CI, confidence interval; GBM, glioblastoma; HR, hazard ratio; OR, odds ratio. Blue squares = effect sizes after excluding one individual study. Gray diamonds = original overall pooled effect sizes.
**Figure S9:** Sensitivity analyses for overall survival benefits of vaccine combination therapy vs. standard therapy in GBM patients. CI, confidence interval; GBM, glioblastoma; HR, hazard ratio; OR, odds ratio. Blue squares = effect sizes after excluding one individual study. Gray diamonds = original overall pooled effect sizes.
**Figure S10:** Comparative analyses of vaccine combination therapy vs. standard therapy in GBM patients (overall population with subgroup of MRD‐assessed patients), stratified by treatment setting. (A) Median progression‐free survival. (B) 6‐month Progression‐free survival rate. (C) 1‐year Progression‐free survival rate. (D) 2‐year Progression‐free survival rate. CI, confidence interval; EGFRvIII, epidermal growth factor receptor variant III; GBM, glioblastoma; HR, hazard ratio; MRD, minimal residual disease; OR, odds ratio. Gray squares = individual study effect sizes (weighted by sample size). Blue diamonds = pooled effect sizes. Horizontal lines = 95% CI.
**Figure S11:** Comparative analyses of vaccine combination therapy vs. standard therapy in GBM patients (overall population with subgroup of MRD‐assessed patients), stratified by treatment setting. (A) Median overall survival. (B) 6‐month Overall survival rate. (C) 1‐year Overall survival rate. (D) 2‐year Overall survival rate. (E) 3‐year Overall survival rate. CI, confidence interval; EGFRvIII, epidermal growth factor receptor variant III; GBM, glioblastoma; HR, hazard ratio; MRD, minimal residual disease; OR, odds ratio. Gray squares = individual study effect sizes (weighted by sample size). Blue diamonds = pooled effect sizes. Horizontal lines = 95% CI.
**Figure S12:** Proportional and sensitivity analyses of overall survival benefits of EGF/EGFR vaccines combination therapy in single‐arm NSCLC and GBM studies, stratified by treatment setting. (A) 6‐month Overall survival rate. (B) 1‐year Overall survival rate. 1 L, 1st‐line; 2 L, 2nd‐line; CI, confidence interval; EGF, epidermal growth factor; EGFRvIII, epidermal growth factor receptor variant III; GBM, glioblastoma; NSCLC, non‐small cell lung cancer. Gray squares = individual study effect sizes (weighted by sample size). Blue diamonds = pooled effect sizes. Horizontal lines = 95% CI. In sensitivity analysis, blue squares = effect sizes after excluding one individual study; gray diamonds = original overall pooled effect sizes.
**Figure S13:** Proportional and sensitivity analyses of progression‐free survival benefits of EGF/EGFR vaccines combination therapy in single‐arm NSCLC and GBM studies, stratified by cancer type. (A) 6‐month Progression‐free survival rate. (B) 1‐year Progression‐free survival rate. 1 L, 1st‐line; 2 L, 2nd‐line; CI, confidence interval; EGF, epidermal growth factor; EGFRvIII, epidermal growth factor receptor variant III; GBM, glioblastoma; NSCLC, non‐small cell lung cancer. Gray squares = individual study effect sizes (weighted by sample size). Blue diamonds = pooled effect sizes. Horizontal lines = 95% CI. In sensitivity analysis, blue squares = effect sizes after excluding one individual study; gray diamonds = original overall pooled effect sizes.
**Figure S14:** Proportional analyses of objective response rate and disease control rate benefits of EGF/EGFR vaccines combination therapy in single‐arm NSCLC studies, stratified by EGFR status. (A) Objective response rate. (B) Disease control rate. NSCLC, non‐small cell lung cancer. 1 L, 1st‐line; 2 L, 2nd‐line; CI, confidence interval; EGF, epidermal growth factor; EGFR, epidermal growth factor receptor; TKI: tyrosine kinase inhibitor. Gray squares = individual study effect sizes (weighted by sample size). Blue diamonds = pooled effect sizes. Horizontal lines = 95% CI.
**Figure S15:** Proportional and sensitivity analyses of overall and grade ≥ 3 adverse events incidence rates of EGF/EGFR vaccines monotherapy, stratified by medicine type. (A) Overall adverse events. (B) Grade ≥ 3 adverse events. CI, confidence interval; EGF, epidermal growth factor; EGFR, epidermal growth factor receptor; NSCLC, non‐small cell lung cancer. Gray squares = individual study effect sizes (weighted by sample size). Blue diamonds = pooled effect sizes. Horizontal lines = 95% CI. In sensitivity analysis, blue squares = effect sizes after excluding one individual study; gray diamonds = original overall pooled effect sizes.
**Figure S16:** Proportional and sensitivity analyses of injection site reactions incidence rates of EGF vaccines monotherapy, stratified by medicine type. (A) Injection site pain. (B) Injection site induration. (C) Injection site erythema. CI, confidence interval; CRC: colorectal cancer; EGF, epidermal growth factor; LC, lung cancer; NSCLC, non‐small cell lung cancer; PC, pancreatic cancer. Gray squares = individual study effect sizes (weighted by sample size). Blue diamonds = pooled effect sizes. Horizontal lines = 95% CI. In sensitivity analysis, blue squares = effect sizes after excluding one individual study; gray diamonds = original overall pooled effect sizes.
**Figure S17:** Proportional and sensitivity analyses of constitutional symptom incidence rates of EGF/EGFR vaccines monotherapy, stratified by medicine type. (A) Chills. (B) Fever. NSCLC, non‐small cell lung cancer; LC, lung cancer; CRC, colorectal cancer; PC, pancreatic cancer; EGF, epidermal growth factor; EGFR, epidermal growth factor receptor; CI, confidence interval. Gray squares = individual study effect sizes (weighted by sample size). Blue diamonds = pooled effect sizes. Horizontal lines = 95% CI. In sensitivity analysis, blue squares = effect sizes after excluding one individual study; gray diamonds = original overall pooled effect sizes.
**Figure S18:** Proportional and sensitivity analyses of nervous system symptom (headache) incidence rate of EGF/EGFR vaccines monotherapy, stratified by medicine type. NSCLC, non‐small cell lung cancer. CI, confidence interval; EGF, epidermal growth factor; EGFRvIII, epidermal growth factor receptor variant III. Gray squares = individual study effect sizes (weighted by sample size). Blue diamonds = pooled effect sizes. Horizontal lines = 95% CI. In sensitivity analysis, blue squares = effect sizes after excluding one individual study; gray diamonds = original overall pooled effect sizes.
**Figure S19:** Proportional and sensitivity analyses of gastrointestinal symptom incidence rates of EGF vaccines monotherapy, stratified by medicine type. (A) Nausea. (B) Vomiting. CI, confidence interval; EGF, epidermal growth factor; NSCLC, non‐small cell lung cancer. Gray squares = individual study effect sizes (weighted by sample size). Blue diamonds = pooled effect sizes. Horizontal lines = 95% CI. In sensitivity analysis, blue squares = effect sizes after excluding one individual study; gray diamonds = original overall pooled effect sizes.

## Data Availability

The data that support the findings of this study are available from the corresponding author upon reasonable request.
