# Supplementary material for: Efficacy and Safety of EGF/EGFR Vaccines in EGFR‐Driven Solid Tumors: A Systematic Review and Meta‐Analysis of Controlled and Single‐Arm Studies
Source: Cancer Med. 2025 Oct 9;14(19):e71295. doi: 10.1002/cam4.71295 (PMC12510904; doi:10.1002/cam4.71295)
Supplement: Supplementary file 1 — Table S1: PRISMA 2020 Checklist. Table S2: Search strategies of possible publications. Table S3: Time‐specific survival rates and calculated HR data digitized from survival curves. Table S4: The most common therapy‐related AEs in vaccine combination therapy. Figure S1: Risk of bias assessment for the included non‐randomized controlled trials and single‐arm studies, according to the MINORS Evaluation Criteria. Figure S2: Risk of bias assessment of randomized controlled trials assessing EGF/EGFR vaccines vs. best supportive care. (A) Risk of bias summary (judgments about each risk of bias item for each included study). (B) Risk of bias graph (judgments about each risk of bias item presented as percentages across all included studies). Figure S3: Risk of bias assessment of randomized controlled trials assessing EGF/EGFR vaccines combination therapy vs. controlled onco‐specific treatment alone. (A) Risk of bias summary (judgments about each risk of bias item for each included study). (B) Risk of bias graph (judgments about each risk of bias item presented as percentages across all included studies). Figure S4: Comparative analyses of EGF/EGFR vaccines vs. best supportive care in NSCLC and GBM patients, stratified by cancer. (A) 6‐month Overall survival rate. (B) 1‐year Overall survival rate. (C) 2‐year Overall survival rate. 1 L, 1st‐line; 2 L, 2nd‐line; CI, confidence interval; EGF, epidermal growth factor; EGFRvIII, epidermal growth factor receptor variant III; GBM, glioblastoma; NSCLC, non‐small cell lung cancer; OR, odds ratio. Gray squares = individual study effect sizes (weighted by sample size). Blue diamonds = pooled effect sizes. Horizontal lines = 95% CI. Figure S5: Sensitivity analyses for pooled benefits of EGF/EGFR vaccines vs. best supportive care in NSCLC and GBM patients. (A) Median overall survival. (B) 6‐month Overall survival rate. (C) 1‐year Overall survival rate. (D) 2‐year Overall survival rate. (E) 3‐year Overall survival rate. 1 L, 1st‐line; 2 L, [file CAM4-14-e71295-s001.docx]

# Supplementary

| **Table S1.** PRISMA 2020 Checklist.  **Table S2.** Search strategies of possible publications.  **Table S3.** Time-specific survival rates and calculated HR data digitized from survival curves.  **Table S4.** The most common therapy-related AEs in vaccine combination therapy.  **Figure S1.** Risk of bias assessment for the included non-randomized controlled trials and single-arm studies, according to the MINORS Evaluation Criteria.  **Figure S2.** Risk of bias assessment of randomized controlled trials assessing EGF/EGFR vaccines vs. best supportive care. (A) Risk of bias summary (judgments about each risk of bias item for each included study). (B) Risk of bias graph (judgements about each risk of bias item presented as percentages across all included studies).  **Figure S3.** Risk of bias assessment of randomized controlled trials assessing EGF/EGFR vaccines combination therapy vs. controlled onco-specific treatment alone. (A) Risk of bias summary (judgments about each risk of bias item for each included study). (B) Risk of bias graph (judgements about each risk of bias item presented as percentages across all included studies).  **Figure S4.** Comparative analyses of EGF/EGFR vaccines vs. best supportive care in NSCLC and GBM patients, stratified by cancer. (A) 6-month Overall survival rate. (B) 1-year Overall survival rate. (C) 2-year Overall survival rate. 1 L, 1st-line; 2 L, 2nd-line; CI, confidence interval; EGF, epidermal growth factor; EGFRvIII, epidermal growth factor receptor variant III; GBM, glioblastoma; NSCLC, non-small cell lung cancer; OR, odds ratio. Gray squares = individual study effect sizes (weighted by sample size). Blue diamonds = pooled effect sizes. Horizontal lines = 95% CI.  **Figure S5.** Sensitivity analyses for pooled benefits of EGF/EGFR vaccines vs. best supportive care in NSCLC and GBM patients. (A) Median overall survival. (B) 6-month Overall survival rate. (C) 1-year Overall survival rate. (D) 2-year Overall survival rate. (E) 3-year Overall survival rate. 1 L, 1st-line; 2 L, 2nd-line; CI, confidence interval; EGF, epidermal growth factor; EGFRvIII, epidermal growth factor receptor variant III; GBM, glioblastoma; NSCLC, non-small cell lung cancer; OR, odds ratio. Blue squares = effect sizes after excluding one individual study. Gray diamonds = original overall pooled effect sizes.  **Figure S6.** Comparative analyses of vaccine combination therapy vs. standard therapy in GBM patients, stratified by treatment setting. (A) Median progression-free survival. (B) 6-month Progression-free survival rate. (C) 1-year Progression-free survival rate. (D) Objective response rate. CI, confidence interval; GBM, glioblastoma; HR, hazard ratio; OR, odds ratio. Gray squares = individual study effect sizes (weighted by sample size). Blue diamonds = pooled effect sizes. Horizontal lines = 95% CI.  **Figure S7.** Comparative analyses of vaccine combination therapy vs. standard therapy in GBM patients, stratified by treatment setting. (A) Median overall survival. (B) 6-month Overall survival rate. (C) 1-year Overall survival rate. (D) 2-year Overall survival rate. CI, confidence interval; GBM, glioblastoma; HR, hazard ratio; OR, odds ratio. Gray squares = individual study effect sizes (weighted by sample size). Blue diamonds = pooled effect sizes. Horizontal lines = 95% CI.  **Figure S8.** Sensitivity analyses for progression-free survival benefits of vaccine combination therapy vs. standard therapy in GBM patients. (A) Median progression-free survival. (B) 6-month Progression-free survival rate. (C) 1-year Progression-free survival rate. (D) 2-year Progression-free survival rate. CI, confidence interval; GBM, glioblastoma; HR, hazard ratio; OR, odds ratio. Blue squares = effect sizes after excluding one individual study. Gray diamonds = original overall pooled effect sizes.  **Figure S9.** Sensitivity analyses for overall survival benefits of vaccine combination therapy vs. standard therapy in GBM patients. (A) Median overall survival. (B) 6-month Overall survival rate. (C) 1-year Overall survival rate. (D) 2-year Overall survival rate. (E) 3-year Overall survival rate. CI, confidence interval; GBM, glioblastoma; HR, hazard ratio; OR, odds ratio. Blue squares = effect sizes after excluding one individual study. Gray diamonds = original overall pooled effect sizes.  **Figure S10.** Comparative analyses of vaccine combination therapy vs. standard therapy in GBM patients (overall population with subgroup of MRD-assessed patients), stratified by treatment setting. (A) Median progression-free survival. (B) 6-month Progression-free survival rate. (C) 1-year Progression-free survival rate. (D) 2-year Progression-free survival rate. CI, confidence interval; EGFRvIII, epidermal growth factor receptor variant III; GBM, glioblastoma; HR, hazard ratio; MRD, minimal residual disease; OR, odds ratio. Gray squares = individual study effect sizes (weighted by sample size). Blue diamonds = pooled effect sizes. Horizontal lines = 95% CI.  **Figure S11.** Comparative analyses of vaccine combination therapy vs. standard therapy in GBM patients (overall population with subgroup of MRD-assessed patients), stratified by treatment setting. (A) Median overall survival. (B) 6-month Overall survival rate. (C) 1-year Overall survival rate. (D) 2-year Overall survival rate. (E) 3-year Overall survival rate. CI, confidence interval; EGFRvIII, epidermal growth factor receptor variant III; GBM, glioblastoma; HR, hazard ratio; MRD, minimal residual disease; OR, odds ratio. Gray squares = individual study effect sizes (weighted by sample size). Blue diamonds = pooled effect sizes. Horizontal lines = 95% CI.  **Figure S12.** Proportional and sensitivity analyses of overall survival benefits of EGF/EGFR vaccines combination therapy in single-arm NSCLC and GBM studies, stratified by treatment setting. (A) 6-month Overall survival rate. (B) 1-year Overall survival rate. 1 L, 1st-line; 2 L, 2nd-line; CI, confidence interval; EGF, epidermal growth factor; EGFRvIII, epidermal growth factor receptor variant III; GBM, glioblastoma; NSCLC, non-small cell lung cancer. Gray squares = individual study effect sizes (weighted by sample size). Blue diamonds = pooled effect sizes. Horizontal lines = 95% CI. In sensitivity analysis, blue squares = effect sizes after excluding one individual study; gray diamonds = original overall pooled effect sizes.  **Figure S13.** Proportional and sensitivity analyses of progression-free survival benefits of EGF/EGFR vaccines combination therapy in single-arm NSCLC and GBM studies, stratified by cancer type. (A) 6-month Progression-free survival rate. (B) 1-year Progression-free survival rate. 1 L, 1st-line; 2 L, 2nd-line; CI, confidence interval; EGF, epidermal growth factor; EGFRvIII, epidermal growth factor receptor variant III; GBM, glioblastoma; NSCLC, non-small cell lung cancer. Gray squares = individual study effect sizes (weighted by sample size). Blue diamonds = pooled effect sizes. Horizontal lines = 95% CI. In sensitivity analysis, blue squares = effect sizes after excluding one individual study; gray diamonds = original overall pooled effect sizes.  **Figure S14.** Proportional analyses of objective response rate and disease control rate benefits of EGF/EGFR vaccines combination therapy in single-arm NSCLC studies, stratified by EGFR status. (A) Objective response rate. (B) Disease control rate. NSCLC, non-small cell lung cancer. 1 L, 1st-line; 2 L, 2ndline; CI, confidence interval; EGF, epidermal growth factor; EGFR, epidermal growth factor receptor; TKI: tyrosine kinase inhibitor. Gray squares = individual study effect sizes (weighted by sample size). Blue diamonds = pooled effect sizes. Horizontal lines = 95% CI.  **Figure S15.** Proportional and sensitivity analyses of overall and grade ≥3 adverse events incidence rates of EGF/EGFR vaccines monotherapy, stratified by medicine type. (A) Overall adverse events. (B) Grade ≥3 adverse events. CI, confidence interval; EGF, epidermal growth factor; EGFR, epidermal growth factor receptor; NSCLC, non-small cell lung cancer. Gray squares = individual study effect sizes (weighted by sample size). Blue diamonds = pooled effect sizes. Horizontal lines = 95% CI. In sensitivity analysis, blue squares = effect sizes after excluding one individual study; gray diamonds = original overall pooled effect sizes.  **Figure S16.** Proportional and sensitivity analyses of injection site reactions incidence rates of EGF vaccines monotherapy, stratified by medicine type. (A) Injection site pain. (B) Injection site induration. (C) Injection site erythema. CI, confidence interval; CRC: colorectal cancer; EGF, epidermal growth factor; LC, lung cancer; NSCLC, non-small cell lung cancer; PC, pancreatic cancer. Gray squares = individual study effect sizes (weighted by sample size). Blue diamonds = pooled effect sizes. Horizontal lines = 95% CI. In sensitivity analysis, blue squares = effect sizes after excluding one individual study; gray diamonds = original overall pooled effect sizes.  **Figure S17.** Proportional and sensitivity analyses of constitutional symptom incidence rates of EGF/EGFR vaccines monotherapy, stratified by medicine type. (A) Chills. (B) Fever. NSCLC, non-small cell lung cancer; LC, lung cancer; CRC, colorectal cancer; PC, pancreatic cancer; EGF, epidermal growth factor; EGFR, epidermal growth factor receptor; CI, confidence interval. Gray squares = individual study effect sizes (weighted by sample size). Blue diamonds = pooled effect sizes. Horizontal lines = 95% CI. In sensitivity analysis, blue squares = effect sizes after excluding one individual study; gray diamonds = original overall pooled effect sizes.  **Figure S18.** Proportional and sensitivity analyses of nervous system symptom (headache) incidence rate of EGF/EGFR vaccines monotherapy, stratified by medicine type. NSCLC, non-small cell lung cancer. CI, confidence interval; EGF, epidermal growth factor; EGFRvIII, epidermal growth factor receptor variant III. Gray squares = individual study effect sizes (weighted by sample size). Blue diamonds = pooled effect sizes. Horizontal lines = 95% CI. In sensitivity analysis, blue squares = effect sizes after excluding one individual study; gray diamonds = original overall pooled effect sizes.  **Figure S19.** Proportional and sensitivity analyses of gastrointestinal symptoms incidence rates of EGF vaccines monotherapy, stratified by medicine type. (A) Nausea. (B) Vomiting. CI, confidence interval; EGF, epidermal growth factor; NSCLC, non-small cell lung cancer. Gray squares = individual study effect sizes (weighted by sample size). Blue diamonds = pooled effect sizes. Horizontal lines = 95% CI. In sensitivity analysis, blue squares = effect sizes after excluding one individual study; gray diamonds = original overall pooled effect sizes. |
| --- |

**Table S1.** PRISMA 2020 Checklist.

| **Section and Topic** | **Item #** | **Checklist item** | **Location where item is reported** |
| --- | --- | --- | --- |
| **TITLE** | | |  |
| Title | 1 | Identify the report as a systematic review. | 1 |
| **ABSTRACT** | | |  |
| Abstract | 2 | See the PRISMA 2020 for Abstracts checklist. | 1-2 |
| **INTRODUCTION** | | |  |
| Rationale | 3 | Describe the rationale for the review in the context of existing knowledge. | 2 |
| Objectives | 4 | Provide an explicit statement of the objective(s) or question(s) the review addresses. | 2 |
| **METHODS** | | |  |
| Eligibility criteria | 5 | Specify the inclusion and exclusion criteria for the review and how studies were grouped for the syntheses. | 2-3 |
| Information sources | 6 | Specify all databases, registers, websites, organisations, reference lists and other sources searched or consulted to identify studies. Specify the date when each source was last searched or consulted. | 3 |
| Search strategy | 7 | Present the full search strategies for all databases, registers and websites, including any filters and limits used. | Supplementary Table S2 |
| Selection process | 8 | Specify the methods used to decide whether a study met the inclusion criteria of the review, including how many reviewers screened each record and each report retrieved, whether they worked independently, and if applicable, details of automation tools used in the process. | 3 |
| Data collection process | 9 | Specify the methods used to collect data from reports, including how many reviewers collected data from each report, whether they worked independently, any processes for obtaining or confirming data from study investigators, and if applicable, details of automation tools used in the process. | 3 |
| Data items | 10a | List and define all outcomes for which data were sought. Specify whether all results that were compatible with each outcome domain in each study were sought (e.g. for all measures, time points, analyses), and if not, the methods used to decide which results to collect. | 3 |
|  | 10b | List and define all other variables for which data were sought (e.g. participant and intervention characteristics, funding sources). Describe any assumptions made about any missing or unclear information. | 3 |
| Study risk of bias assessment | 11 | Specify the methods used to assess risk of bias in the included studies, including details of the tool(s) used, how many reviewers assessed each study and whether they worked independently, and if applicable, details of automation tools used in the process. | 3 |
| Effect measures | 12 | Specify for each outcome the effect measure(s) (e.g. risk ratio, mean difference) used in the synthesis or presentation of results. | 3 |
| Synthesis methods | 13a | Describe the processes used to decide which studies were eligible for each synthesis (e.g. tabulating the study intervention characteristics and comparing against the planned groups for each synthesis (item #5)). | 3 |
|  | 13b | Describe any methods required to prepare the data for presentation or synthesis, such as handling of missing summary statistics, or data conversions. | 3 |
|  | 13c | Describe any methods used to tabulate or visually display results of individual studies and syntheses. | 3 |
|  | 13d | Describe any methods used to synthesize results and provide a rationale for the choice(s). If meta-analysis was performed, describe the model(s), method(s) to identify the presence and extent of statistical heterogeneity, and software package(s) used. | 3 |
|  | 13e | Describe any methods used to explore possible causes of heterogeneity among study results (e.g. subgroup analysis, meta-regression). | 7 |
|  | 13f | Describe any sensitivity analyses conducted to assess robustness of the synthesized results. | 7 |
| Reporting bias assessment | 14 | Describe any methods used to assess risk of bias due to missing results in a synthesis (arising from reporting biases). | * |
| Certainty assessment | 15 | Describe any methods used to assess certainty (or confidence) in the body of evidence for an outcome. | 7 |
| **RESULTS** | | |  |
| Study selection | 16a | Describe the results of the search and selection process, from the number of records identified in the search to the number of studies included in the review, ideally using a flow diagram. | 8 |
|  | 16b | Cite studies that might appear to meet the inclusion criteria, but which were excluded, and explain why they were excluded. | 8 |
| Study characteristics | 17 | Cite each included study and present its characteristics. | 4-8 |
| Risk of bias in studies | 18 | Present assessments of risk of bias for each included study. | 8, Supplementary Figures S1-S3 |
| Results of individual studies | 19 | For all outcomes, present, for each study: (a) summary statistics for each group (where appropriate) and (b) an effect estimate and its precision (e.g. confidence/credible interval), ideally using structured tables or plots. | 8-18 |
| Results of syntheses | 20a | For each synthesis, briefly summarise the characteristics and risk of bias among contributing studies. | 8-18 |
|  | 20b | Present results of all statistical syntheses conducted. If meta-analysis was done, present for each the summary estimate and its precision (e.g. confidence/credible interval) and measures of statistical heterogeneity. If comparing groups, describe the direction of the effect. | 8-18 |
|  | 20c | Present results of all investigations of possible causes of heterogeneity among study results. | 8-18 |
|  | 20d | Present results of all sensitivity analyses conducted to assess the robustness of the synthesized results. | 8-18 |
| Reporting biases | 21 | Present assessments of risk of bias due to missing results (arising from reporting biases) for each synthesis assessed. | * |
| Certainty of evidence | 22 | Present assessments of certainty (or confidence) in the body of evidence for each outcome assessed. | 8-18 |
| **DISCUSSION** | | |  |
| Discussion | 23a | Provide a general interpretation of the results in the context of other evidence. | 19 |
|  | 23b | Discuss any limitations of the evidence included in the review. | 21 |
|  | 23c | Discuss any limitations of the review processes used. | 21 |
|  | 23d | Discuss implications of the results for practice, policy, and future research. | 19-21 |
| **OTHER INFORMATION** | | |  |
| Registration and protocol | 24a | Provide registration information for the review, including register name and registration number, or state that the review was not registered. | 2 |
|  | 24b | Indicate where the review protocol can be accessed, or state that a protocol was not prepared. | 2 |
|  | 24c | Describe and explain any amendments to information provided at registration or in the protocol. | - |
| Support | 25 | Describe sources of financial or non-financial support for the review, and the role of the funders or sponsors in the review. | 1 |
| Competing interests | 26 | Declare any competing interests of review authors. | 21 |
| Availability of data, code and other materials | 27 | Report which of the following are publicly available and where they can be found: template data collection forms; data extracted from included studies; data used for all analyses; analytic code; any other materials used in the review. | 21 |

*From:*  Page MJ, McKenzie JE, Bossuyt PM, Boutron I, Hoffmann TC, Mulrow CD, et al. The PRISMA 2020 statement: an updated guideline for reporting systematic reviews. BMJ 2021;372:n71. doi: 10.1136/bmj.n71. This work is licensed under CC BY 4.0. To view a copy of this license, visit <https://creativecommons.org/licenses/by/4.0/>

*: Publication bias assessment (funnel plots, Egger’s test) was not performed due to insufficient studies, below the recommended threshold for such analyses.

**Table S2.** Literature search criteria.

| PubMed: 658 citations |
| --- |
| ((((((((((((((((Neoplasms[MeSH Terms]) OR (Tumors[Title/Abstract])) OR (Neoplasia[Title/Abstract])) OR (Neoplasias[Title/Abstract])) OR (Neoplasm[Title/Abstract])) OR (Tumor[Title/Abstract])) OR (Cancer[Title/Abstract])) OR (Cancers[Title/Abstract])) OR ("Malignant Neoplasm"[Title/Abstract])) OR (Malignancy[Title/Abstract])) OR (Malignancies[Title/Abstract])) OR ("Malignant Neoplasms"[Title/Abstract])) OR ("Neoplasm, Malignant"[Title/Abstract])) OR ("Neoplasms, Malignant"[Title/Abstract])) AND ((((((((((((("ErbB Receptors"[MeSH Terms]) OR ("ErbB Receptors"[Title/Abstract])) OR ("epidermal growth factor receptor"[Title/Abstract])) OR (EGFR[Title/Abstract])) OR ("Epidermal Growth Factor Receptor Variant III"[Title/Abstract])) OR ("Epidermal Growth Factor Receptor vIII"[Title/Abstract])) OR ("EGFR variant III"[Title/Abstract])) OR (EGFRvIII[Title/Abstract])) OR (EGFR-vIII[Title/Abstract])) OR (EGFRΔIII[Title/Abstract])) OR (EGFRdeltaIII[Title/Abstract])) OR ("Epidermal Growth Factor"[Title/Abstract])) OR (EGF[Title/Abstract]))) AND (((((Vaccines[MeSH Terms]) OR (Vaccines[Title/Abstract])) OR (Vaccine[Title/Abstract])) OR (Vaccination[Title/Abstract])) OR (Vaccin*[Title/Abstract]))) AND (((Patients[MeSH Terms]) OR (Patients[Title/Abstract])) OR (Patient[Title/Abstract])) |
| Embase: 1418 citations |
| #1 'malignant neoplasm'/exp OR tumors:ab,ti,kw OR neoplasia:ab,ti,kw OR neoplasias:ab,ti,kw OR neoplasm:ab,ti,kw OR tumor:ab,ti,kw OR cancer:ab,ti,kw OR cancers:ab,ti,kw OR 'malignant neoplasm':ab,ti,kw OR malignancy:ab,ti,kw OR malignancies:ab,ti,kw OR 'malignant neoplasms':ab,ti,kw OR 'neoplasm, malignant':ab,ti,kw OR 'neoplasms, malignant':ab,ti,kw  #2 'epidermal growth factor receptor'/exp OR 'epidermal growth factor receptor':ab,ti,kw OR 'erbb receptors':ab,ti,kw OR egfr:ab,ti,kw OR 'epidermal growth factor receptor variant iii':ti,ab,kw OR 'epidermal growth factor receptor viii':ti,ab,kw OR 'egfr variant iii':ti,ab,kw OR egfrviii:ti,ab,kw OR 'egfr-viii':ti,ab,kw OR 'egfrδiii':ti,ab,kw OR 'egfrdeltaiii':ti,ab,kw OR 'epidermal growth factor':ti,ab,kw OR egf:ab,ti,kw  #3 vaccine/exp OR vaccine:ab,ti,kw OR vaccines:ab,ti,kw OR vaccination:ab,ti,kw OR vaccin*:ab,ti,kw  #4 'patient'/exp OR patient:ab,ti,kw OR patients:ab,ti,kw  #5 #1 AND #2 AND #3 AND #4 |
| Cochrane Central Register of Controlled Trials: 140 citations |
| #1 MeSH descriptor: [Neoplasms] explode all trees  #2 (Neoplasms):ti,ab,kw OR (Tumors):ti,ab,kw OR (Neoplasia):ti,ab,kw OR (Neoplasias):ti,ab,kw OR (Neoplasm):ti,ab,kw  #3 (Tumor):ti,ab,kw OR (Cancer):ti,ab,kw OR (Cancers):ti,ab,kw OR ("Malignant Neoplasm"):ti,ab,kw OR (Malignancy):ti,ab,kw  #4 (Malignancies):ti,ab,kw OR ("Malignant Neoplasms"):ti,ab,kw OR ("Neoplasm, Malignant"):ti,ab,kw OR ("Neoplasms, Malignant"):ti,ab,kw  #5 #1 OR #2 OR #3 OR #4  #6 MeSH descriptor: [ErbB Receptors] explode all trees  #7 MeSH descriptor: [Epidermal Growth Factor] explode all trees  #8 ("ErbB Receptors"):ti,ab,kw OR ("epidermal growth factor receptor"):ti,ab,kw OR (EGFR):ti,ab,kw OR ("Epidermal Growth Factor"):ti,ab,kw OR (EGF):ti,ab,kw  #9 ("Epidermal Growth Factor Receptor Variant III"):ti,ab,kw OR ("Epidermal Growth Factor Receptor vIII"):ti,ab,kw OR ("EGFR variant III"):ti,ab,kw OR (EGFRvIII):ti,ab,kw OR (EGFR-vIII):ti,ab,kw  #10 (EGFRΔIII):ti,ab,kw OR (EGFRdeltaIII):ti,ab,kw  #11 #6 OR #7 OR #8 OR #9 OR #10  #12 MeSH descriptor: [Vaccines] explode all trees  #13 (Vaccines):ti,ab,kw OR (Vaccine):ti,ab,kw OR (Vaccination):ti,ab,kw OR (Vaccin*):ti,ab,kw  #14 #12 OR #13  #15 MeSH descriptor: [Patients] explode all trees  #16 (Patients):ti,ab,kw OR (Patient):ti,ab,kw  #17 #15 OR #16  #18 #5 AND #11 AND #14 AND #17 |

**From inception to July 16, 2025.**

**Table S3.** Time-specific survival rates and calculated HR data digitized from survival curves.

| **Study** | **Outcomes** | | | | | | | | | |
| --- | --- | --- | --- | --- | --- | --- | --- | --- | --- | --- |
|  | **6mPFS (%)** | **1yPFS (%)** | **2yPFS (%)** | **6mOS (%)** | **1yOS (%)** | **2yOS (%)** | **3yOS (%)** | **4yOS (%)** | **5yOS (%)** | **HR**  **(95% CI) for Median OS** |
| **Vaccine monotherapy vs. best supportive care** | | | | | | | | | | |
| Rodriguez 2016 | NR | NR | NR | 78.7 vs. 69.7 | 51.9 vs. 42.9 | 30.8 vs. 25.8 | 21 vs.12.9 | 17.4 vs. 8.2 | ✓ | ✓ |
| Neninger, 2008 | NR | NR | NR | 51.3 vs. 43.4 | 29.4 vs. 13.5 | 16.6 vs. 11.4 | 11.3 vs. 3.9 | NR | NR | 0.70  (0.44-1.12) |
| Sampson, 2010 | NR | NR | NR | ✓ | ✓ | ✓ | 33 vs. 6 | 22 vs. 0 | 22 vs. 0 | ✓ |
| **Vaccine monotherapy** | | | | | | | | | | |
| Flores, 2023 | NR | NR | NR | ✓ | ✓ | ✓ | 19.8 | NR | NR | - |
| **Vaccine combination therapy vs. standard therapy alone** | | | | | | | | | | |
| Reardon, 2020 | ✓ | 18.9 vs. 10.5 | 14 vs. 0 | 86 vs. 75.6 | 44.4 vs. 31.5 | ✓ | 10 vs. 2 | NR | NR | ✓ |
| Weller, 2017 | 51.3 vs. 47.1 | 28 vs. 27.7 | 14.2 vs. 10.4 | 93 vs. 93.5 | 71.6 vs. 72.6 | 32.9 vs. 30 | 19.7 vs. 9.9 | NR | NR | ✓ |
| Sampson, 2011 | ✓ | ✓ | ✓ | ✓ | ✓ | ✓ | 32.9 vs. 5.9 | NR | NR | ✓ |
| **Vaccine combination therapy** | | | | | | | | | | |
| Suarez, 2022 | ✓ | ✓ | NR | ✓ | ✓ | 49.3 | NR | NR | NR | - |
| Rodríguez, 2022 | 95.2 | 78.6 | 31.5 | 100 | 95.2 | 70.5 | 48.8 | NR | NR | - |
| Schuster, 2015 | 68.4 | 38.1 | 12.6 | 96.9 | 86.1 | 46.1 | 26.1 | 13.8 | 13.8 | - |
| Neninger, 2009 | NR | NR | NR | 89.5 | 68.3 | 21 | 14 | NR | NR | - |

The data listed were digitized from the survival curves of the individual included studies. 6mPFS=6-month progression-free survival; 1yPFS=1-year progression-free survival; 2yPFS=2-year progression-free survival; 6mOS=6-month overall survival; 1yOS=1-year overall survival; 2yOS=2-year overall survival; 3yOS=3-year overall survival; 4yOS=4-year overall survival; 5yOS=5-year overall survival; HR=hazard ratio; CI=confidence interval; NR=not reported. ✓: Data reported in included study, which are presented in Table 1. -: Not applicable.

**TableS4.** The most common therapy-related AEs in vaccine combination therapy.

| **Study** | **Target** | **Regimen** | **Most common** **therapy-related AEs (TRAEs)** |
| --- | --- | --- | --- |
| Rodríguez, 2022 (EPICAL) | EGF | hu-recEGF/recP64k + afatinib | Grade 1-2 skin toxicity, diarrhea, paronychia and mucositis. |
| Neninger, 2019 | EGF | CIMAvax-EGF + chemotherapy | Injection-site reaction (7.8%), cough (7.3%), anemia (6.1%), fever (5.3%), asthenia (4.9%) and anorexia (4.9%) (most of them were classified as grade 1-2). |
| Andric, 2018 | EGF | CIMAvax-EGF + chemotherapy | Most patients had nausea and vomiting most likely due to chemotherapy rather than vaccine, other AEs were local reaction, fever, diarrhea, anemia, face erythema, myalgia. |
| Neninger, 2009 | EGF | CIMAvax-EGF + chemotherapy | Related to EGF vaccine were chills (50%), fatigue (45%), nausea/vomiting (45%), arthralgia (30%), and injection site pain (25%); Chemotherapy-associated toxicity included nausea/vomiting (70%) and neuropathy in 5 patients (25%). |
| Ills, 2010 | EGF | CIMAvax-EGF + chemotherapy | Bone pain was the most frequent AE (26.8%) for vaccinated patients. Other frequent AE were vomiting, fever and headache. |
| Suárez, 2022 | EGF | CIMAvax-EGF + thymic polypeptide fraction | Grade 1-2 injection site pain, fever, nausea and headache. |
| Evans, 2022 | EGF | CIMAvax-EGF + nivolumab | Grade 1-2 injection site pain (38.5%), fever (15.4%), vomiting (15.4%). One case of grade 3 myocarditis was attributed to nivolumab. |
| Reardon, 2020 (ReACT) | EGFRvIII | Rindopepimut + bevacizumab | Grade 1–2 injection site reaction (primarily erythema and pruritus) occurred in the majority of patients. |
| Weller, 2017 (ACT IV) | EGFRvIII | Rindopepimut + TMZ | Injection site reactions, consisting chiefly of transient grade 1-2 erythema, pruritus, and rash, were experienced by the majority of the patients who received rindopepimut (80%). |
| Schuster, 2015 | EGFRvIII | Rindopepimut + TMZ | Grade 1-2 injection site reactions, chiefly erythema and pruritus. |
| Liang, 2025  (ABOR2013  -101-GYF)-Part 2 | EGFR | ABOR2013 + sintilimab | Majority of TRAEs were grade 1 or grade 2，the most frequently occurring TEAEs were pyrexia (80%), followed by lymphocyte count decreased (60%), and headache (60%). One case of grade ≥3 TRAEs was related to sintilimab. |
| Wang, 2024 | EGFR | EGFR vaccine + tislelizumab + chemotherapy | Grade 1-2 TRAEs are consistent with known immuno and chemotherapy, while no ≥3 grade AEs were observed. |

EGF=epidermal growth factor. EGFR= epidermal growth factor receptor. EGFRvIII=epidermal growth factor receptor variant III. TMZ= temozolomide.

| 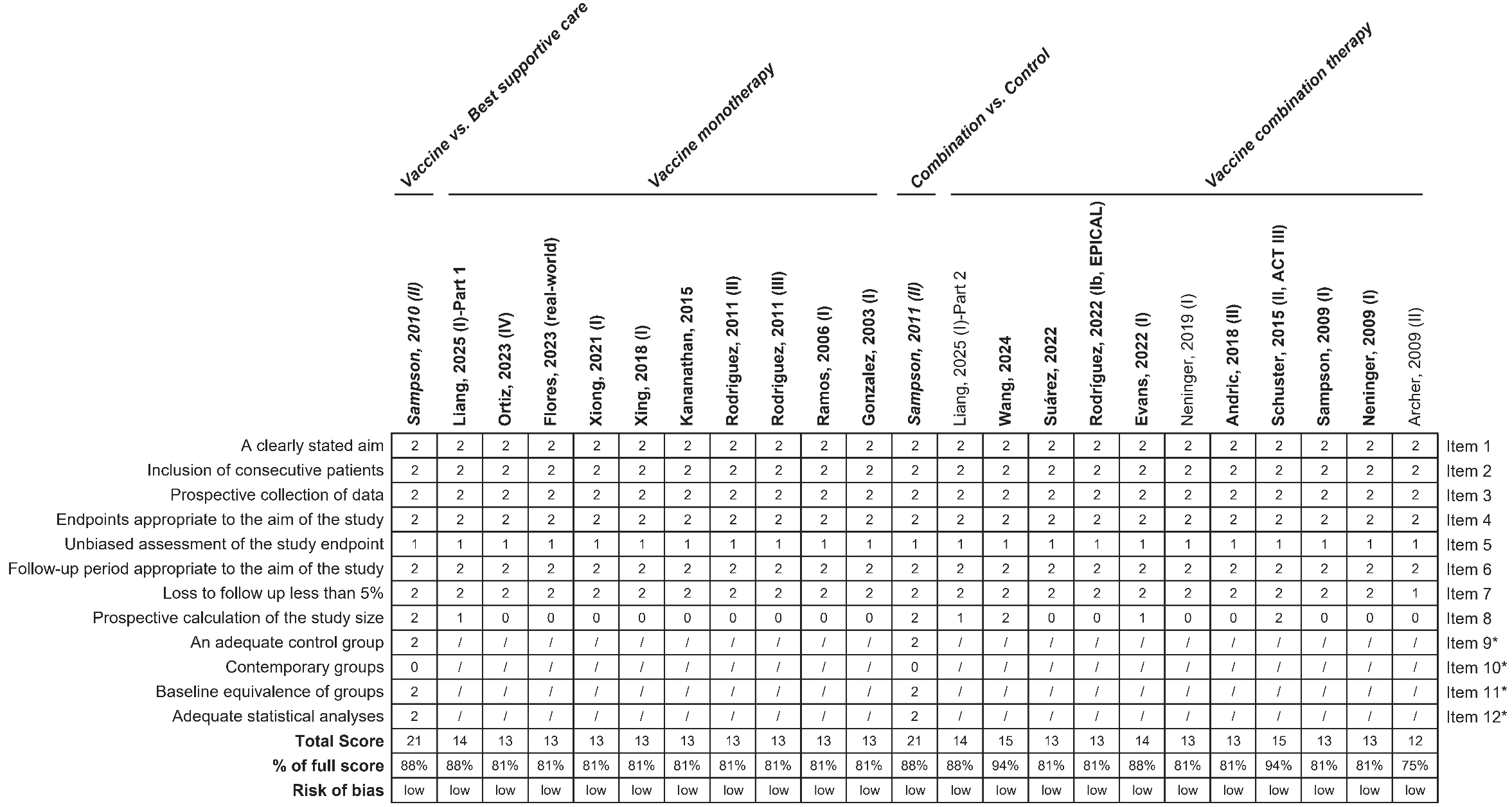 |
| --- |
| **Figure S1.** Risk of bias assessment for the included non-randomized controlled trials and single-arm studies, according to the MINORS Evaluation Criteria. MINORS=Methodological index for non-randomized studies. Studies that are included in both the systematic review and the meta-analysis are indicated in bold, while those included only in the systematic review are not bolded. *: For comparative studies only. Scores of ≥75% were considered high quality with low risk for bias; scores between 50% and 75% were considered medium risk for bias; scores of ≤50% were considered high risk for bias. For noncomparative studies, the maximum score was 16, while the maximum score for comparative studies was 24. |

| 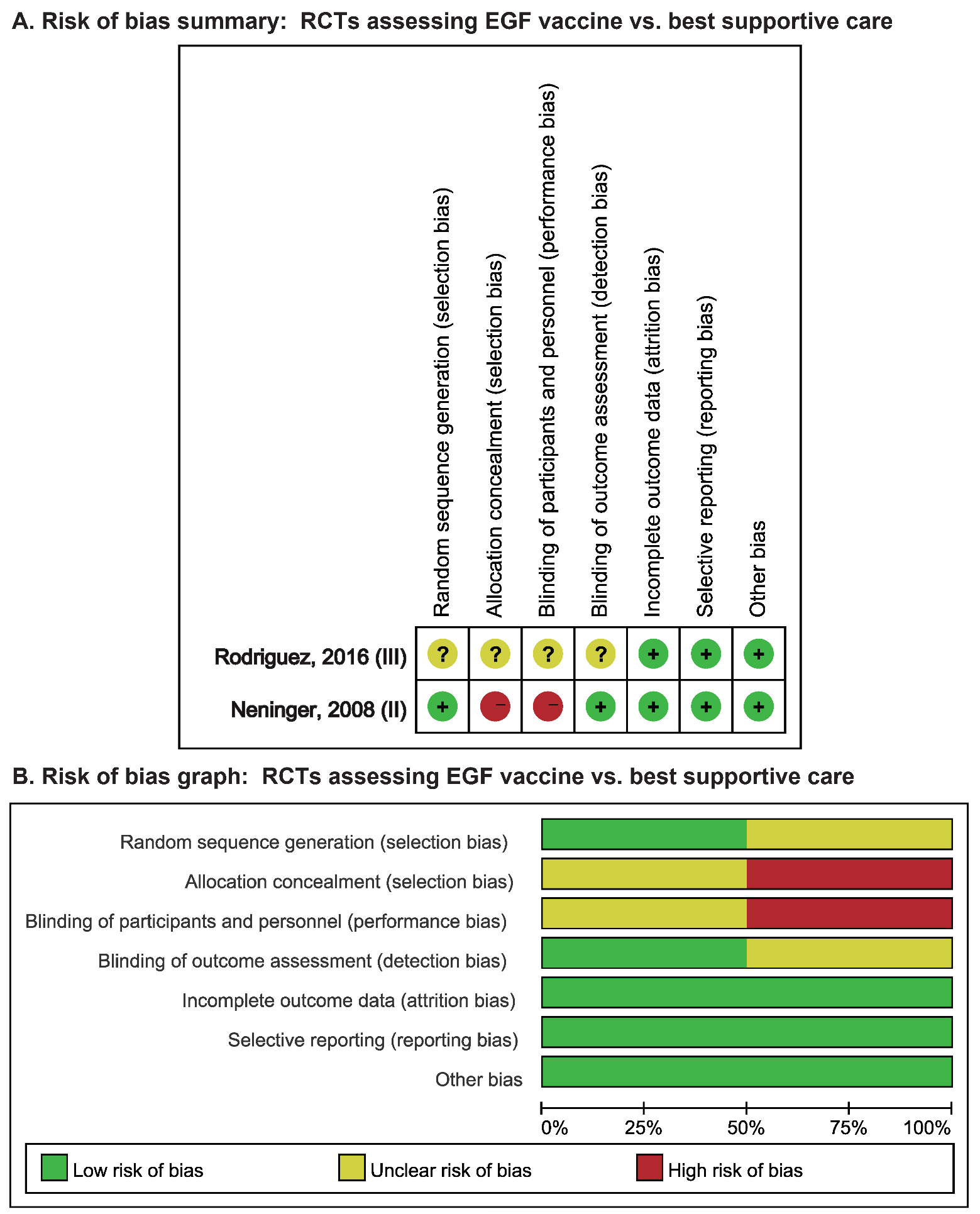 |
| --- |
| **Figure S2.** Risk of bias assessment of randomized controlled trials assessing EGF/EGFR vaccines vs. best supportive care. (A) Risk of bias summary (judgments about each risk of bias item for each included study). (B) Risk of bias graph (judgements about each risk of bias item presented as percentages across all included studies). |

| 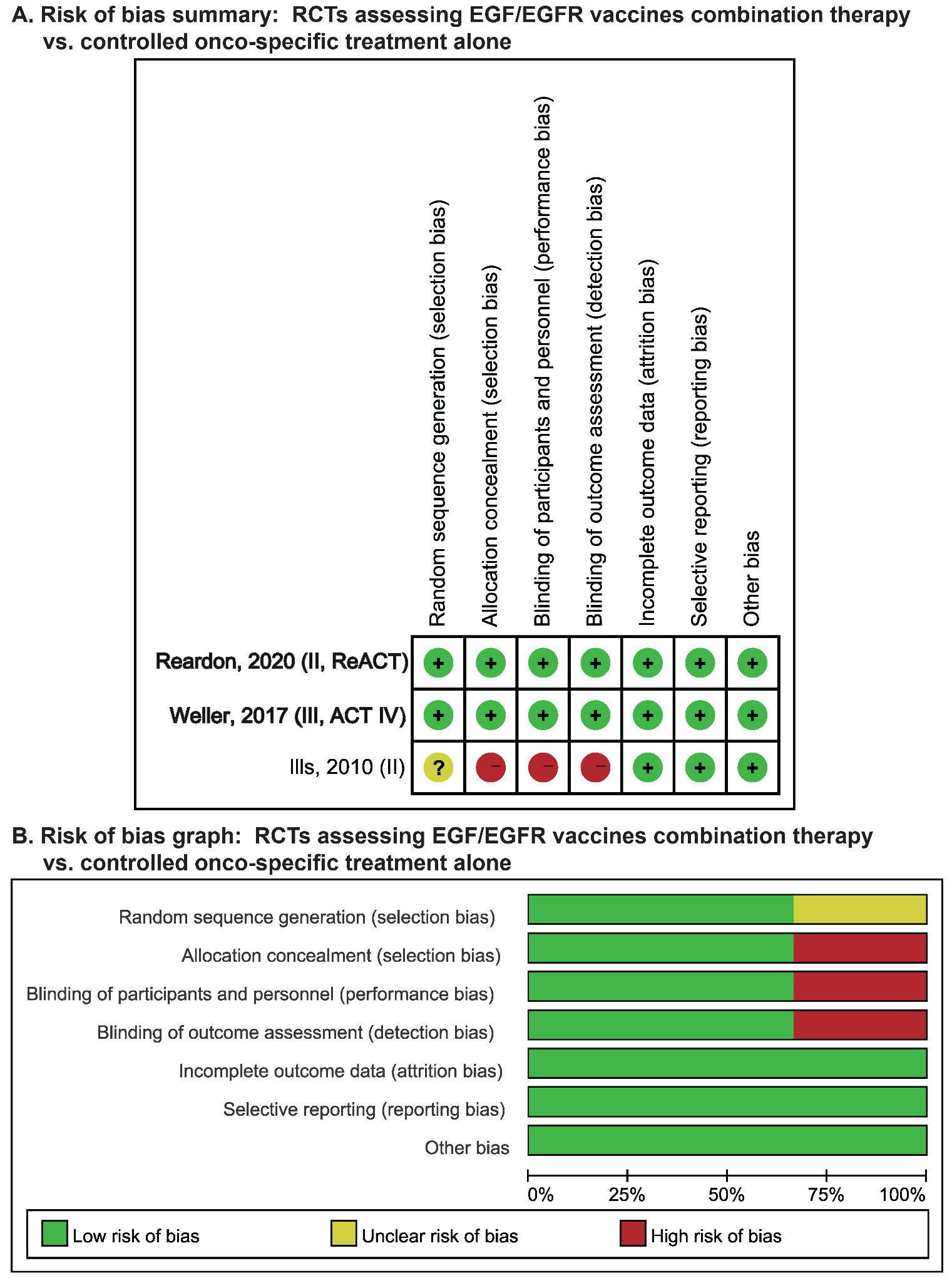 |
| --- |
| **Figure S3.** Risk of bias assessment of randomized controlled trials assessing EGF/EGFR vaccines combination therapy vs. controlled onco-specific treatment alone. (A) Risk of bias summary (judgments about each risk of bias item for each included study). (B) Risk of bias graph (judgements about each risk of bias item presented as percentages across all included studies). |

| 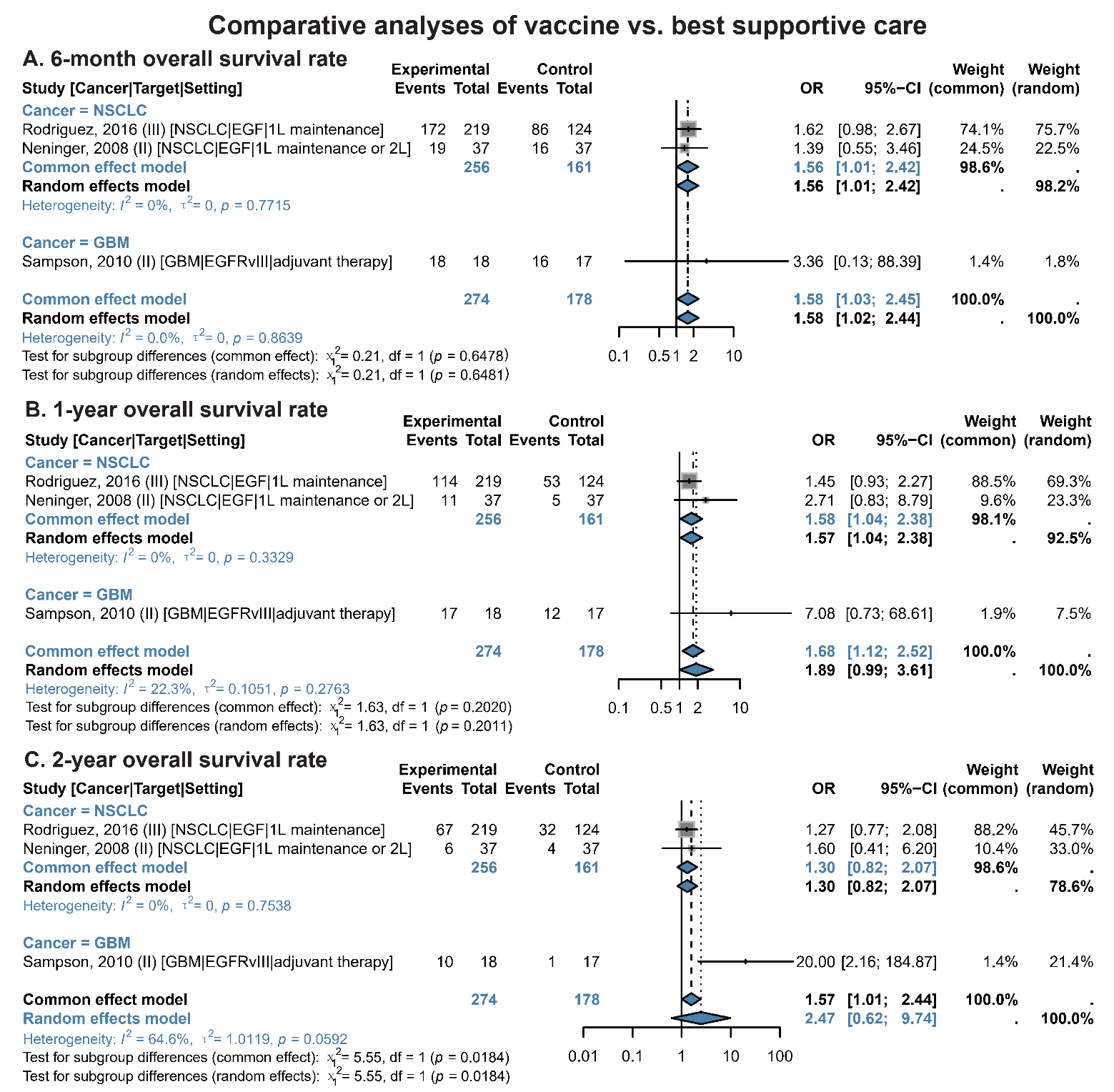 |
| --- |
| **Figure S4.** Comparative analyses of EGF/EGFR vaccines vs. best supportive care in NSCLC and GBM patients, stratified by cancer. (A) 6-month Overall survival rate. (B) 1-year Overall survival rate. (C) 2-year Overall survival rate. 1 L, 1st-line; 2 L, 2nd-line; CI, confidence interval; EGF, epidermal growth factor; EGFRvIII, epidermal growth factor receptor variant III; GBM, glioblastoma; NSCLC, non-small cell lung cancer; OR, odds ratio. Gray squares = individual study effect sizes (weighted by sample size). Blue diamonds = pooled effect sizes. Horizontal lines = 95% CI. |

| 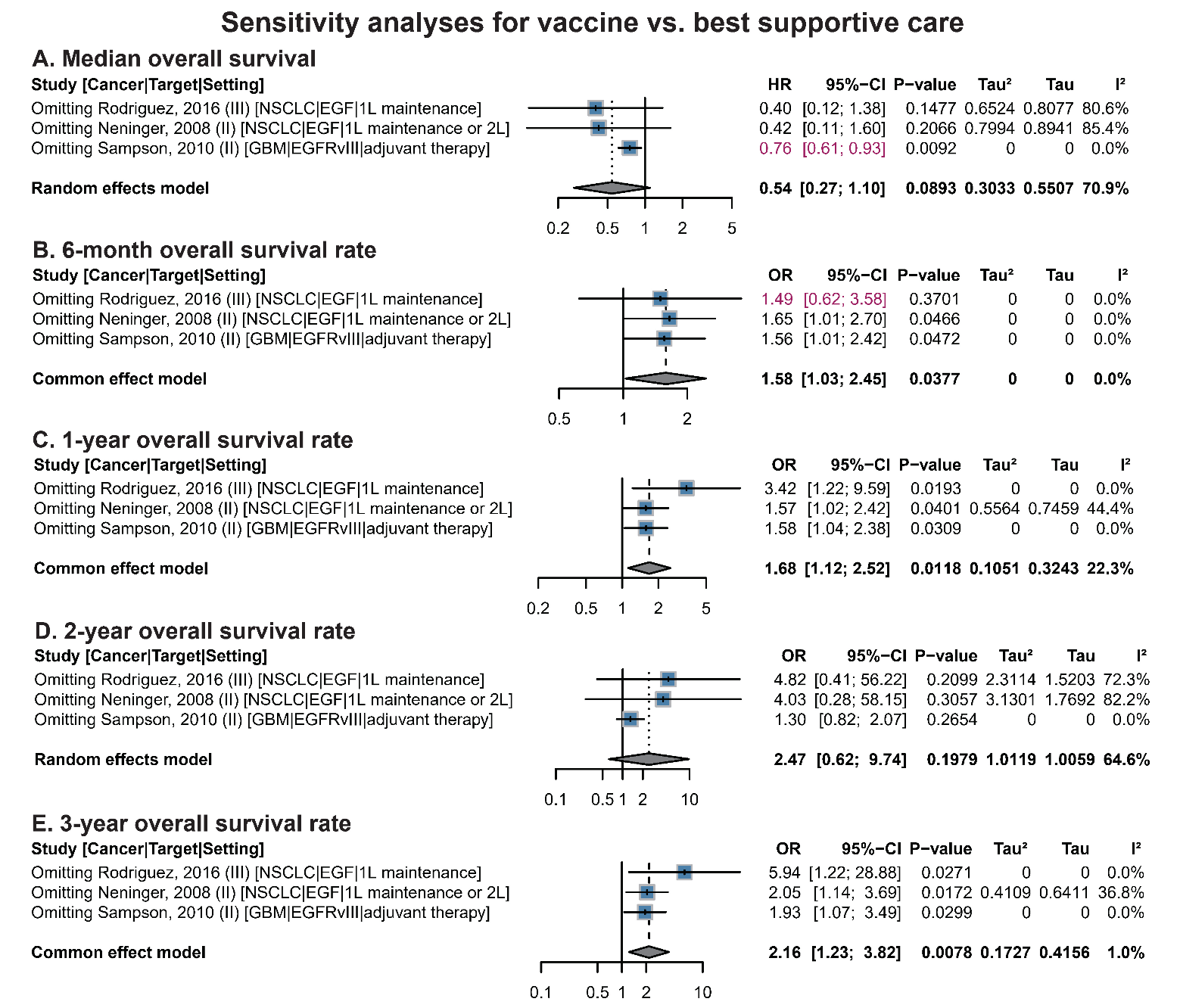 |
| --- |
| **Figure S5.** Sensitivity analyses for pooled benefits of EGF/EGFR vaccines vs. best supportive care in NSCLC and GBM patients. (A) Median overall survival. (B) 6-month Overall survival rate. (C) 1-year Overall survival rate. (D) 2-year Overall survival rate. (E) 3-year Overall survival rate. 1 L, 1st-line; 2 L, 2nd-line; CI, confidence interval; EGF, epidermal growth factor; EGFRvIII, epidermal growth factor receptor variant III; GBM, glioblastoma; NSCLC, non-small cell lung cancer; OR, odds ratio. Blue squares = effect sizes after excluding one individual study. Gray diamonds = original overall pooled effect sizes. |

| 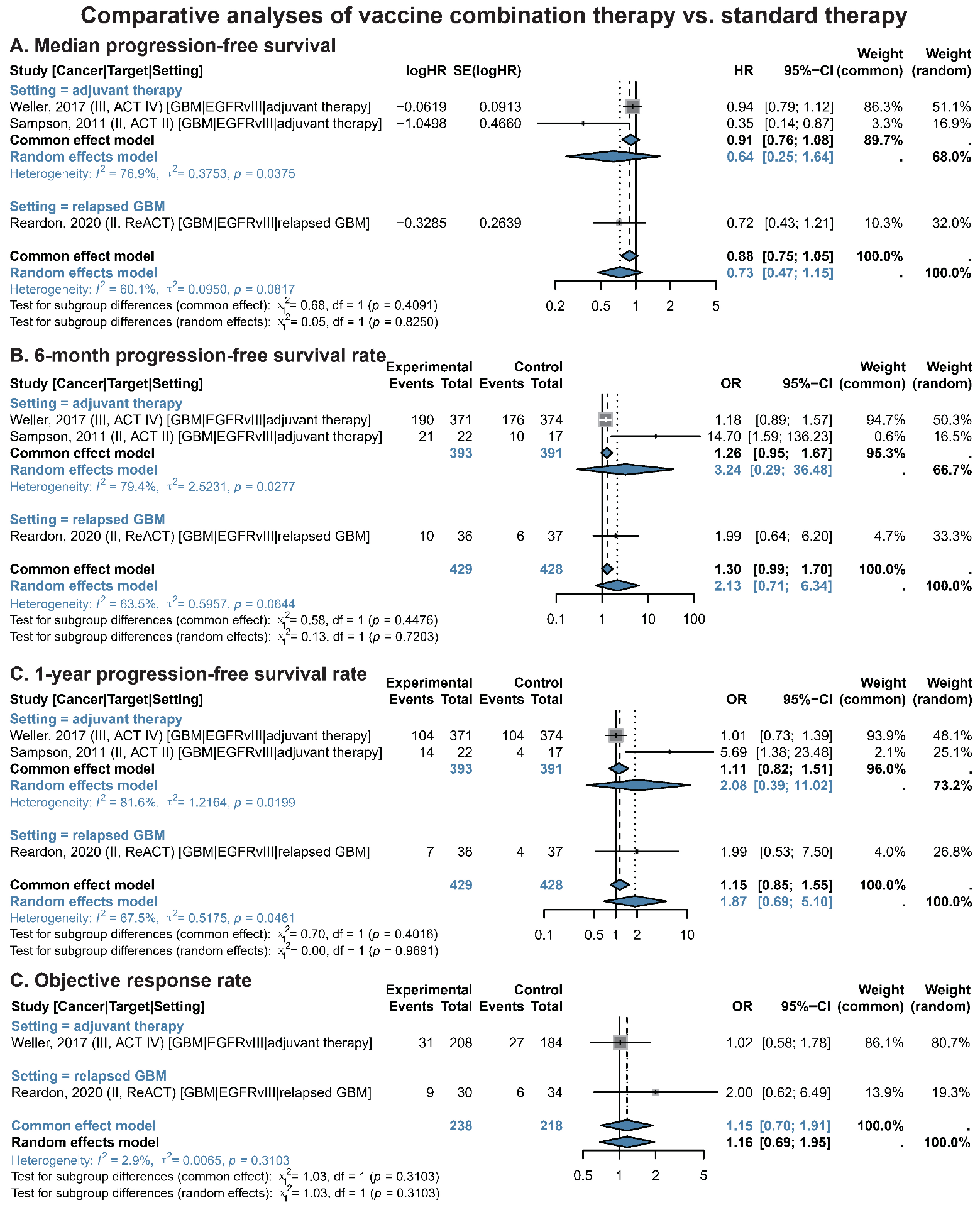 |
| --- |
| **Figure S6.** Comparative analyses of vaccine combination therapy vs. standard therapy in GBM patients, stratified by treatment setting. (A) Median progression-free survival. (B) 6-month Progression-free survival rate. (C) 1-year Progression-free survival rate. (D) Objective response rate. CI, confidence interval; GBM, glioblastoma; HR, hazard ratio; OR, odds ratio. Gray squares = individual study effect sizes (weighted by sample size). Blue diamonds = pooled effect sizes. Horizontal lines = 95% CI. |

| 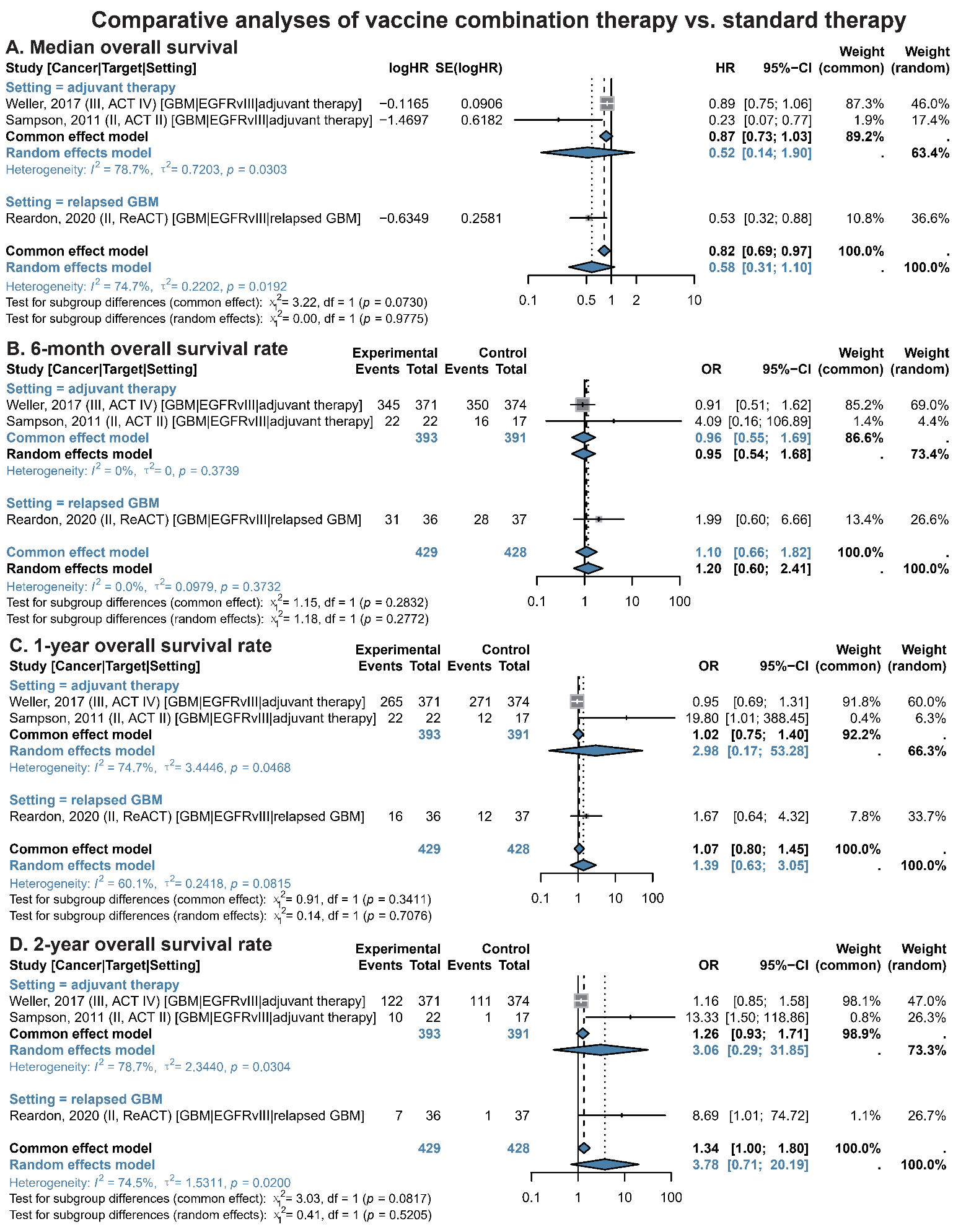 |
| --- |
| **Figure S7.** Comparative analyses of vaccine combination therapy vs. standard therapy in GBM patients, stratified by treatment setting. (A) Median overall survival. (B) 6-month Overall survival rate. (C) 1-year Overall survival rate. (D) 2-year Overall survival rate. CI, confidence interval; GBM, glioblastoma; HR, hazard ratio; OR, odds ratio. Gray squares = individual study effect sizes (weighted by sample size). Blue diamonds = pooled effect sizes. Horizontal lines = 95% CI. |

| 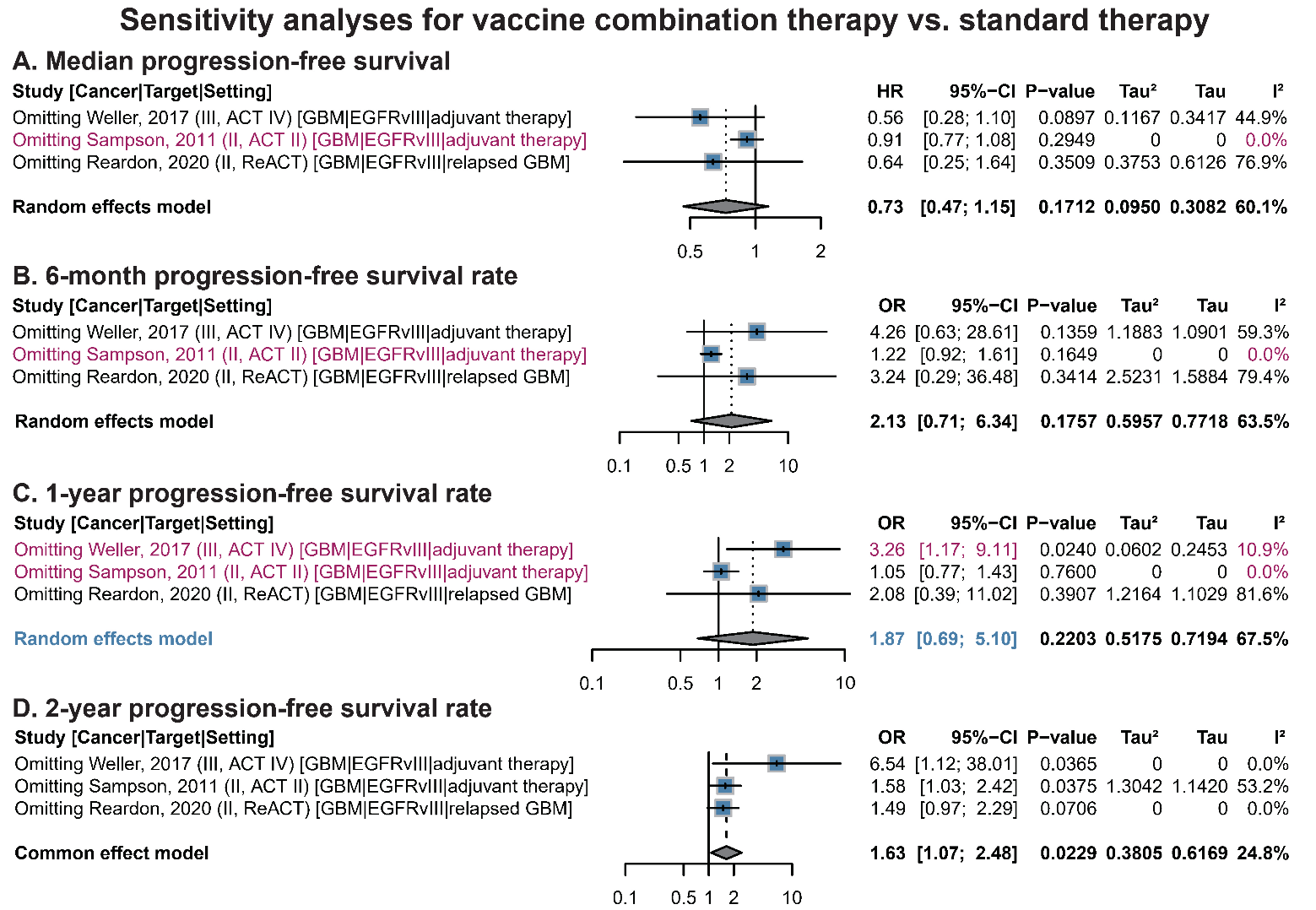 |
| --- |
| **Figure S8.** Sensitivity analyses for progression-free survival benefits of vaccine combination therapy vs. standard therapy in GBM patients. (A) Median progression-free survival. (B) 6-month Progression-free survival rate. (C) 1-year Progression-free survival rate. (D) 2-year Progression-free survival rate. CI, confidence interval; GBM, glioblastoma; HR, hazard ratio; OR, odds ratio. Blue squares = effect sizes after excluding one individual study. Gray diamonds = original overall pooled effect sizes. |

| 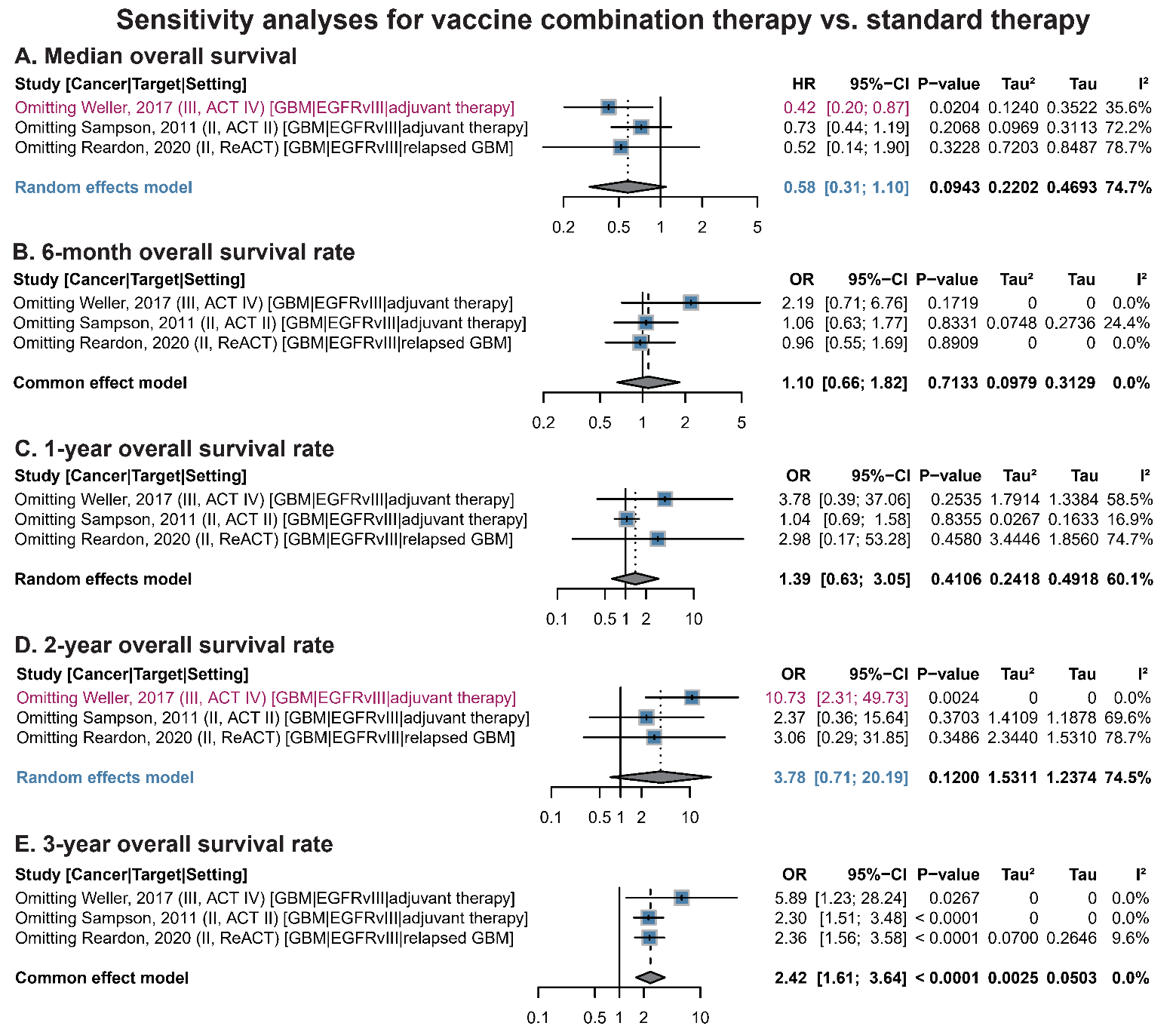 |
| --- |
| **Figure S9.** Sensitivity analyses for overall survival benefits of vaccine combination therapy vs. standard therapy in GBM patients. (A) Median overall survival. (B) 6-month Overall survival rate. (C) 1-year Overall survival rate. (D) 2-year Overall survival rate. (E) 3-year Overall survival rate. CI, confidence interval; GBM, glioblastoma; HR, hazard ratio; OR, odds ratio. Blue squares = effect sizes after excluding one individual study. Gray diamonds = original overall pooled effect sizes. |

| 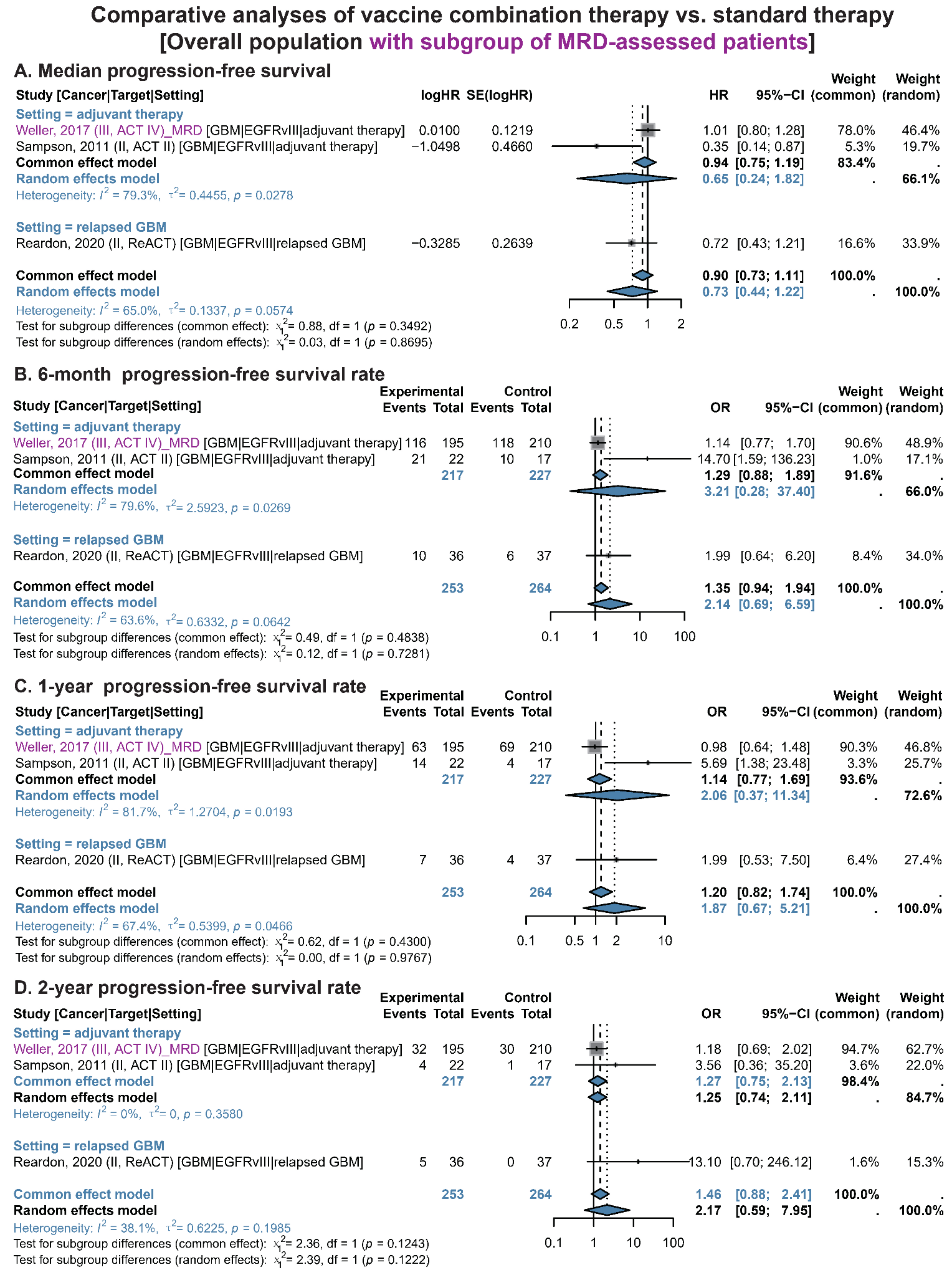 |
| --- |
| **Figure S10.** Comparative analyses of vaccine combination therapy vs. standard therapy in GBM patients (overall population with subgroup of MRD-assessed patients), stratified by treatment setting. (A) Median progression-free survival. (B) 6-month Progression-free survival rate. (C) 1-year Progression-free survival rate. (D) 2-year Progression-free survival rate. CI, confidence interval; EGFRvIII, epidermal growth factor receptor variant III; GBM, glioblastoma; HR, hazard ratio; MRD, minimal residual disease; OR, odds ratio. Gray squares = individual study effect sizes (weighted by sample size). Blue diamonds = pooled effect sizes. Horizontal lines = 95% CI. |
| 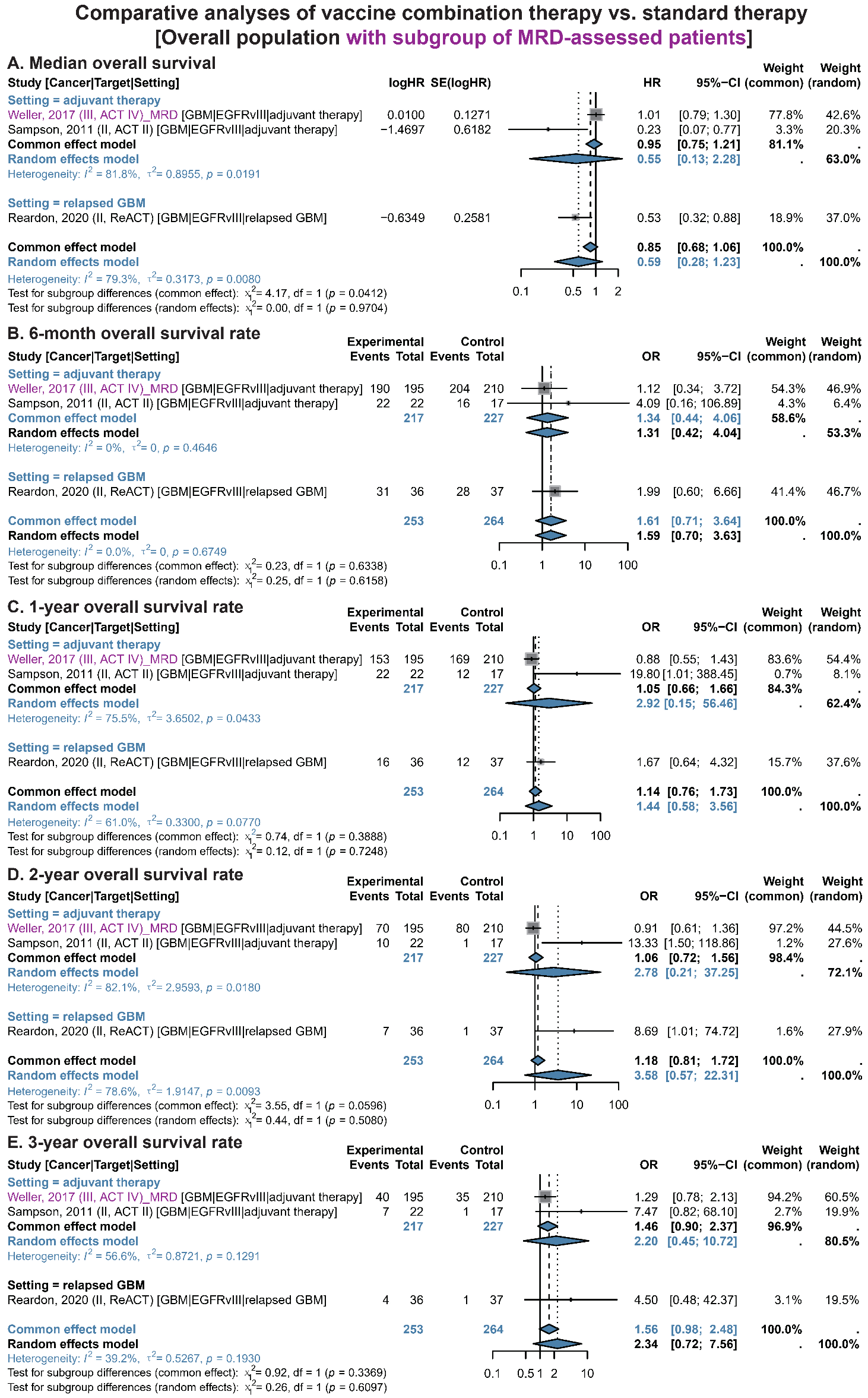 |
| **Figure S11.** Comparative analyses of vaccine combination therapy vs. standard therapy in GBM patients (overall population with subgroup of MRD-assessed patients), stratified by treatment setting. (A) Median overall survival. (B) 6-month Overall survival rate. (C) 1-year Overall survival rate. (D) 2-year Overall survival rate. (E) 3-year Overall survival rate. CI, confidence interval; EGFRvIII, epidermal growth factor receptor variant III; GBM, glioblastoma; HR, hazard ratio; MRD, minimal residual disease; OR, odds ratio. Gray squares = individual study effect sizes (weighted by sample size). Blue diamonds = pooled effect sizes. Horizontal lines = 95% CI. |

| 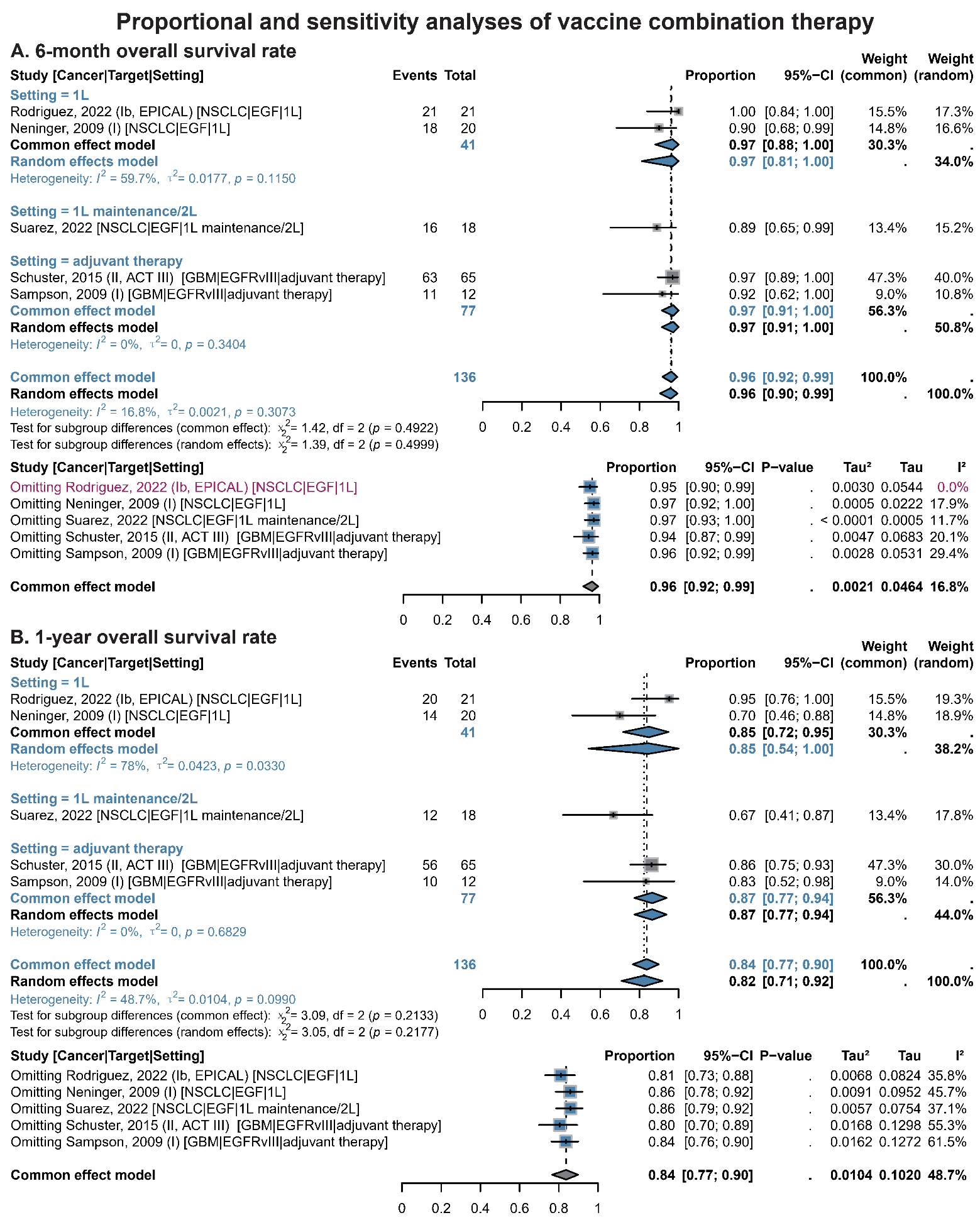 |
| --- |
| **Figure S12.** Proportional and sensitivity analyses of overall survival benefits of EGF/EGFR vaccines combination therapy in single-arm NSCLC and GBM studies, stratified by treatment setting. (A) 6-month Overall survival rate. (B) 1-year Overall survival rate. 1 L, 1st-line; 2 L, 2nd-line; CI, confidence interval; EGF, epidermal growth factor; EGFRvIII, epidermal growth factor receptor variant III; GBM, glioblastoma; NSCLC, non-small cell lung cancer. Gray squares = individual study effect sizes (weighted by sample size). Blue diamonds = pooled effect sizes. Horizontal lines = 95% CI. In sensitivity analysis, blue squares = effect sizes after excluding one individual study; gray diamonds = original overall pooled effect sizes. |
| 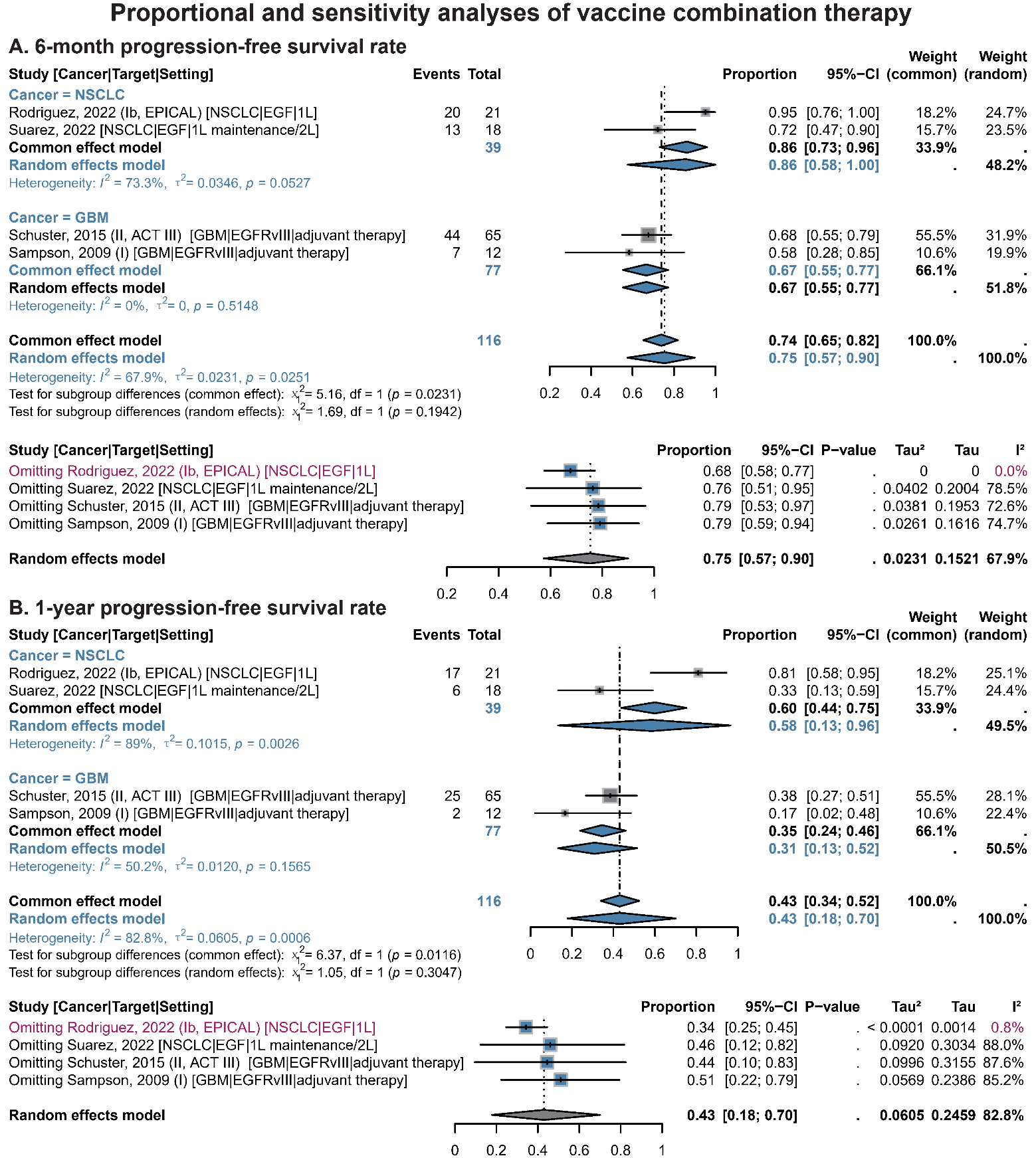 |
| **Figure S13.** Proportional and sensitivity analyses of progression-free survival benefits of EGF/EGFR vaccines combination therapy in single-arm NSCLC and GBM studies, stratified by cancer type. (A) 6-month Progression-free survival rate. (B) 1-year Progression-free survival rate. 1 L, 1st-line; 2 L, 2nd-line; CI, confidence interval; EGF, epidermal growth factor; EGFRvIII, epidermal growth factor receptor variant III; GBM, glioblastoma; NSCLC, non-small cell lung cancer. Gray squares = individual study effect sizes (weighted by sample size). Blue diamonds = pooled effect sizes. Horizontal lines = 95% CI. In sensitivity analysis, blue squares = effect sizes after excluding one individual study; gray diamonds = original overall pooled effect sizes. |

| 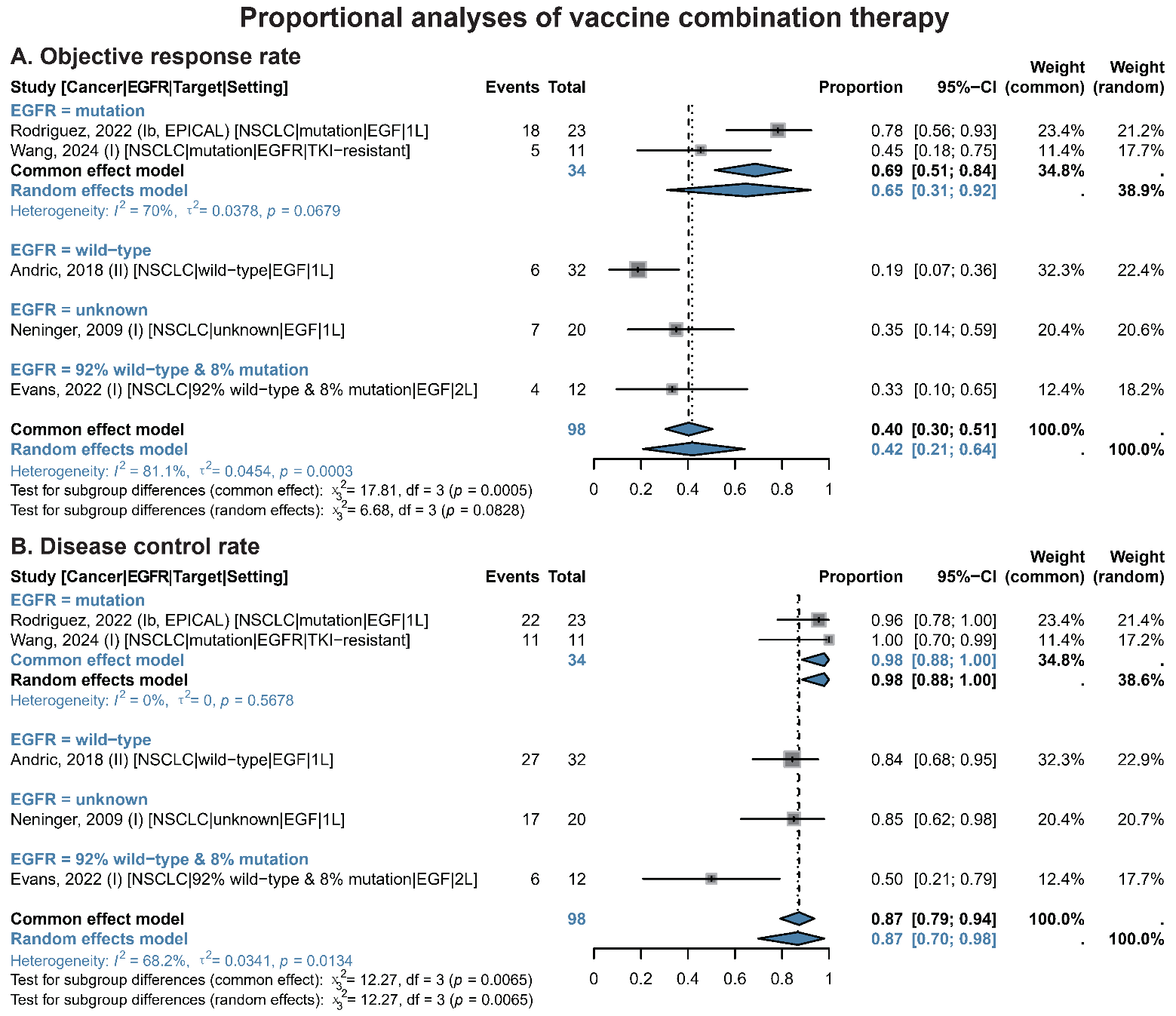 |
| --- |
| **Figure S14.** Proportional analyses of objective response rate and disease control rate benefits of EGF/EGFR vaccines combination therapy in single-arm NSCLC studies, stratified by EGFR status. (A) Objective response rate. (B) Disease control rate. NSCLC, non-small cell lung cancer. 1 L, 1st-line; 2 L, 2ndline; CI, confidence interval; EGF, epidermal growth factor; EGFR, epidermal growth factor receptor; TKI: tyrosine kinase inhibitor. Gray squares = individual study effect sizes (weighted by sample size). Blue diamonds = pooled effect sizes. Horizontal lines = 95% CI. |

| 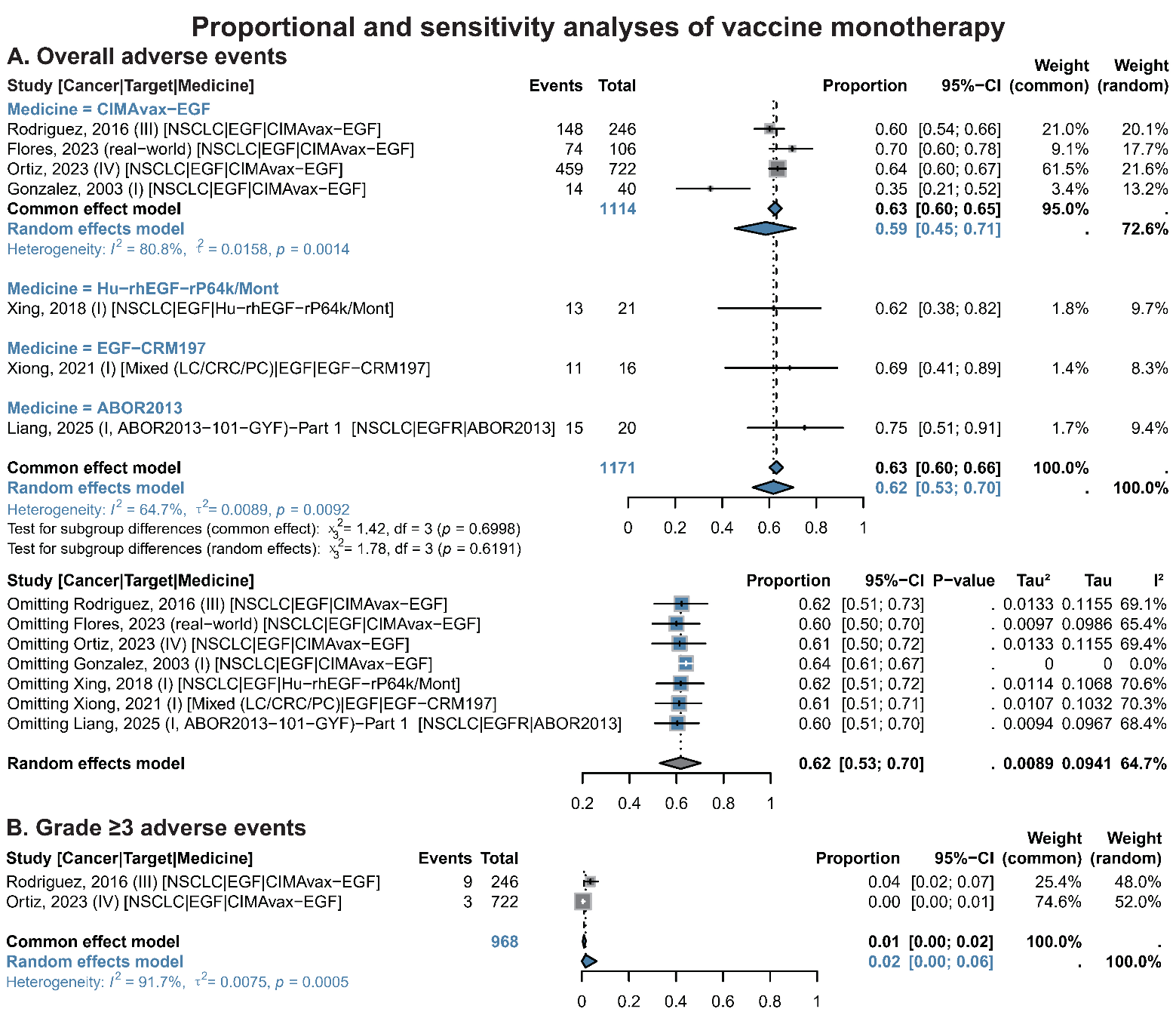 |
| --- |
| **Figure S15.** Proportional and sensitivity analyses of overall and grade ≥3 adverse events incidence rates of EGF/EGFR vaccines monotherapy, stratified by medicine type. (A) Overall adverse events. (B) Grade ≥3 adverse events. CI, confidence interval; EGF, epidermal growth factor; EGFR, epidermal growth factor receptor; NSCLC, non-small cell lung cancer. Gray squares = individual study effect sizes (weighted by sample size). Blue diamonds = pooled effect sizes. Horizontal lines = 95% CI. In sensitivity analysis, blue squares = effect sizes after excluding one individual study; gray diamonds = original overall pooled effect sizes. |

| 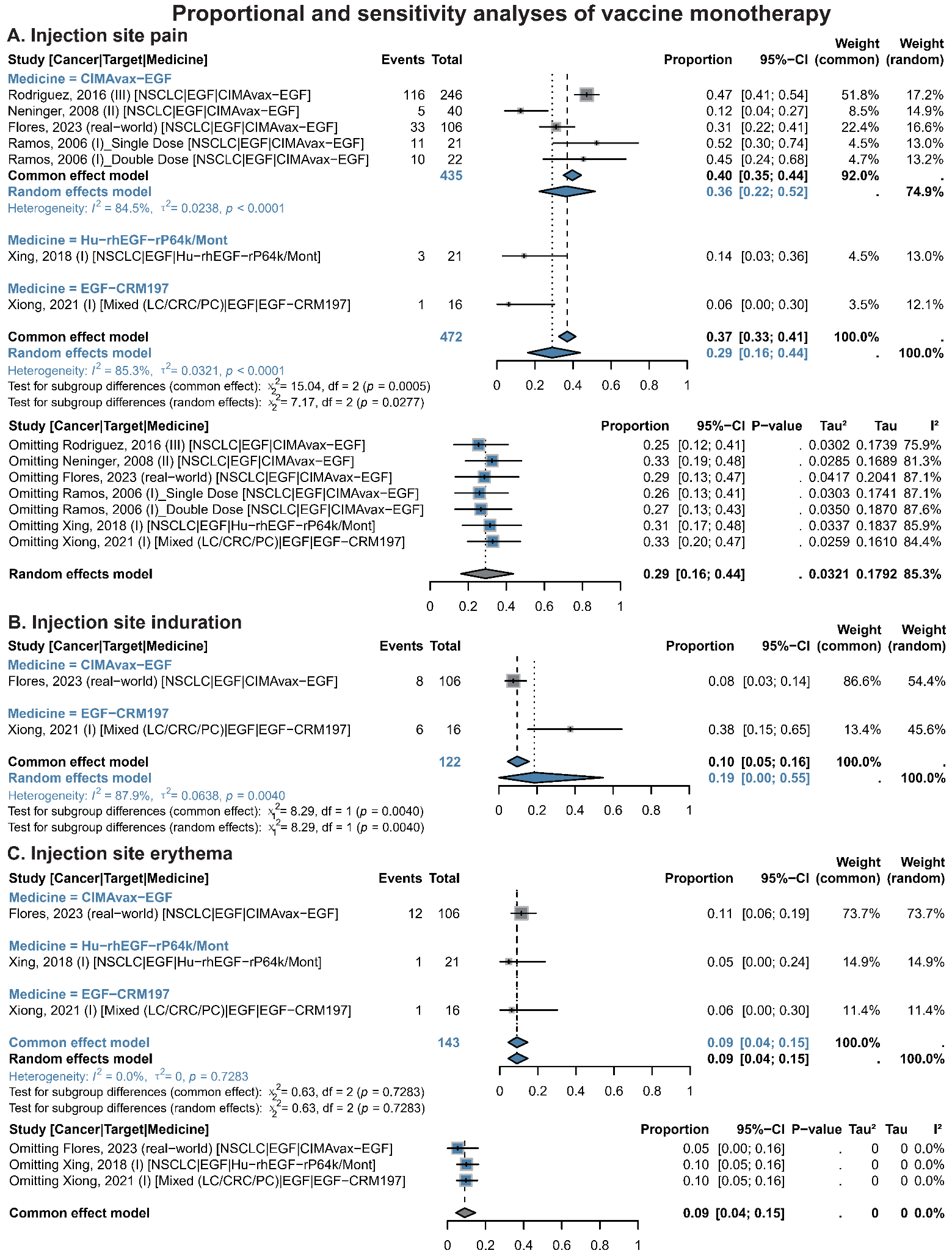 |
| --- |
| **Figure S16.** Proportional and sensitivity analyses of injection site reactions incidence rates of EGF vaccines monotherapy, stratified by medicine type. (A) Injection site pain. (B) Injection site induration. (C) Injection site erythema. CI, confidence interval; CRC: colorectal cancer; EGF, epidermal growth factor; LC, lung cancer; NSCLC, non-small cell lung cancer; PC, pancreatic cancer. Gray squares = individual study effect sizes (weighted by sample size). Blue diamonds = pooled effect sizes. Horizontal lines = 95% CI. In sensitivity analysis, blue squares = effect sizes after excluding one individual study; gray diamonds = original overall pooled effect sizes. |

| 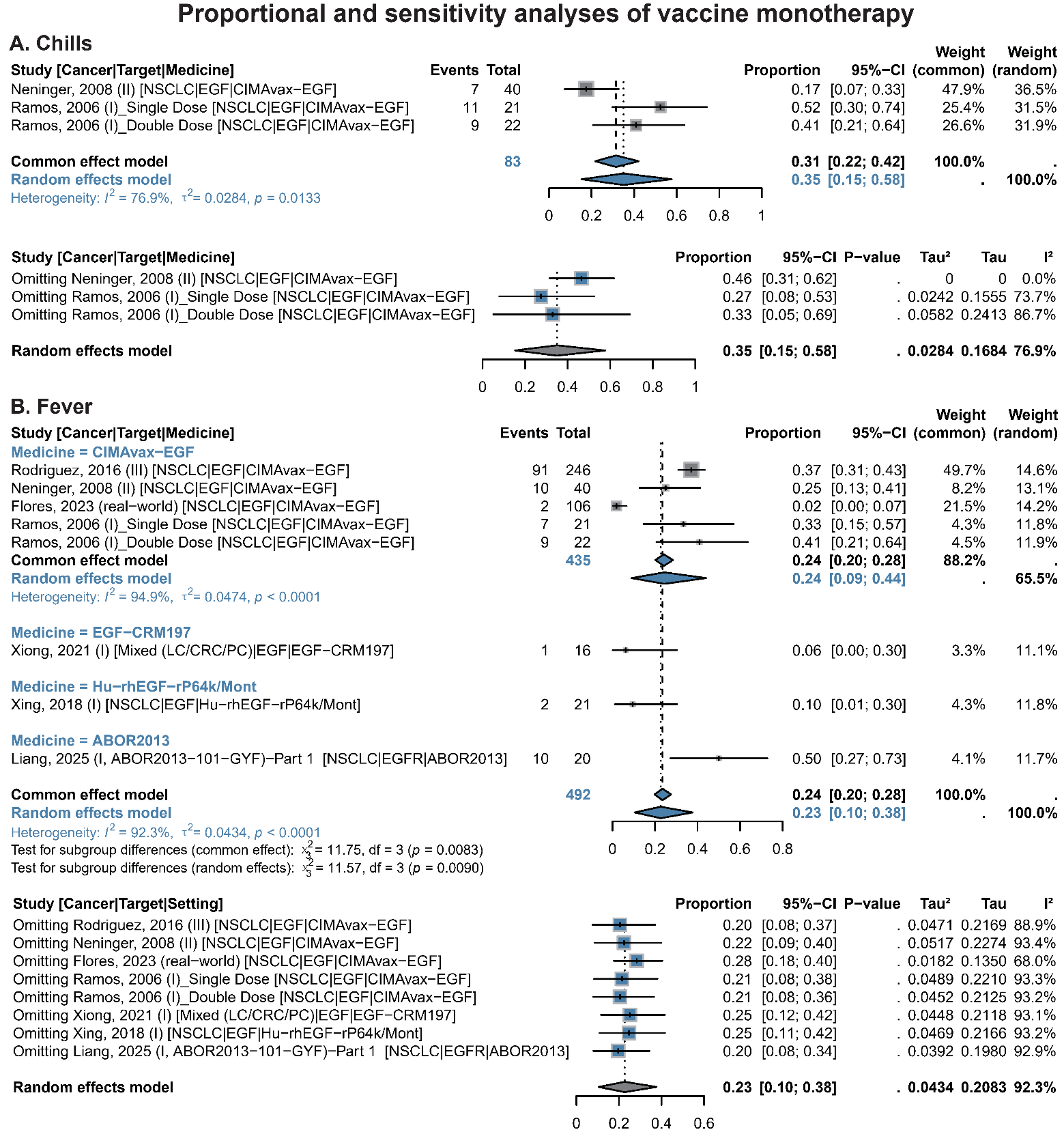 |
| --- |
| **Figure S17.** Proportional and sensitivity analyses of constitutional symptom incidence rates of EGF/EGFR vaccines monotherapy, stratified by medicine type. (A) Chills. (B) Fever. NSCLC, non-small cell lung cancer; LC, lung cancer; CRC, colorectal cancer; PC, pancreatic cancer; EGF, epidermal growth factor; EGFR, epidermal growth factor receptor; CI, confidence interval. Gray squares = individual study effect sizes (weighted by sample size). Blue diamonds = pooled effect sizes. Horizontal lines = 95% CI. In sensitivity analysis, blue squares = effect sizes after excluding one individual study; gray diamonds = original overall pooled effect sizes. |

| 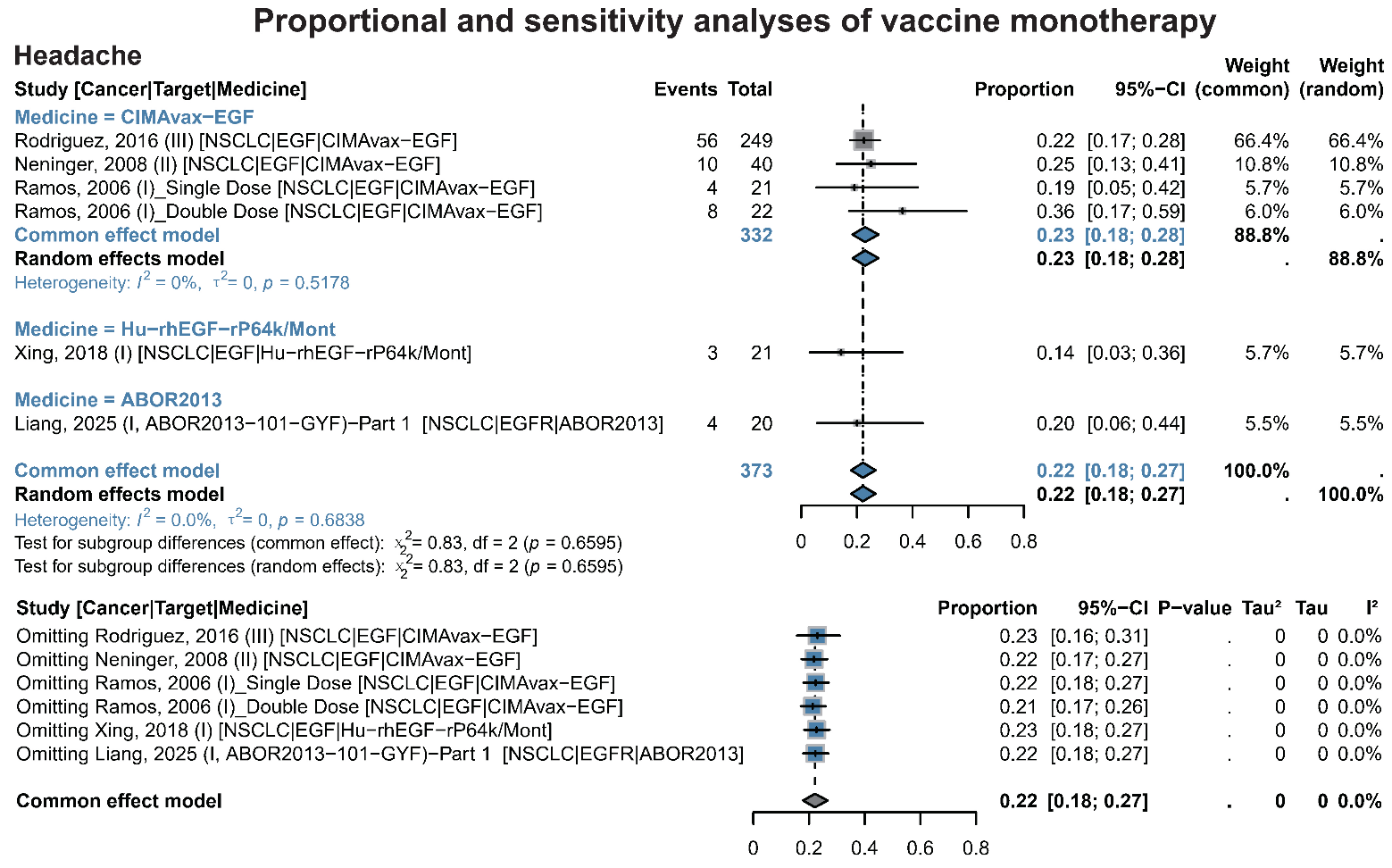 |
| --- |
| **Figure S18.** Proportional and sensitivity analyses of nervous system symptom (headache) incidence rate of EGF/EGFR vaccines monotherapy, stratified by medicine type. NSCLC, non-small cell lung cancer. CI, confidence interval; EGF, epidermal growth factor; EGFRvIII, epidermal growth factor receptor variant III. Gray squares = individual study effect sizes (weighted by sample size). Blue diamonds = pooled effect sizes. Horizontal lines = 95% CI. In sensitivity analysis, blue squares = effect sizes after excluding one individual study; gray diamonds = original overall pooled effect sizes. |

| 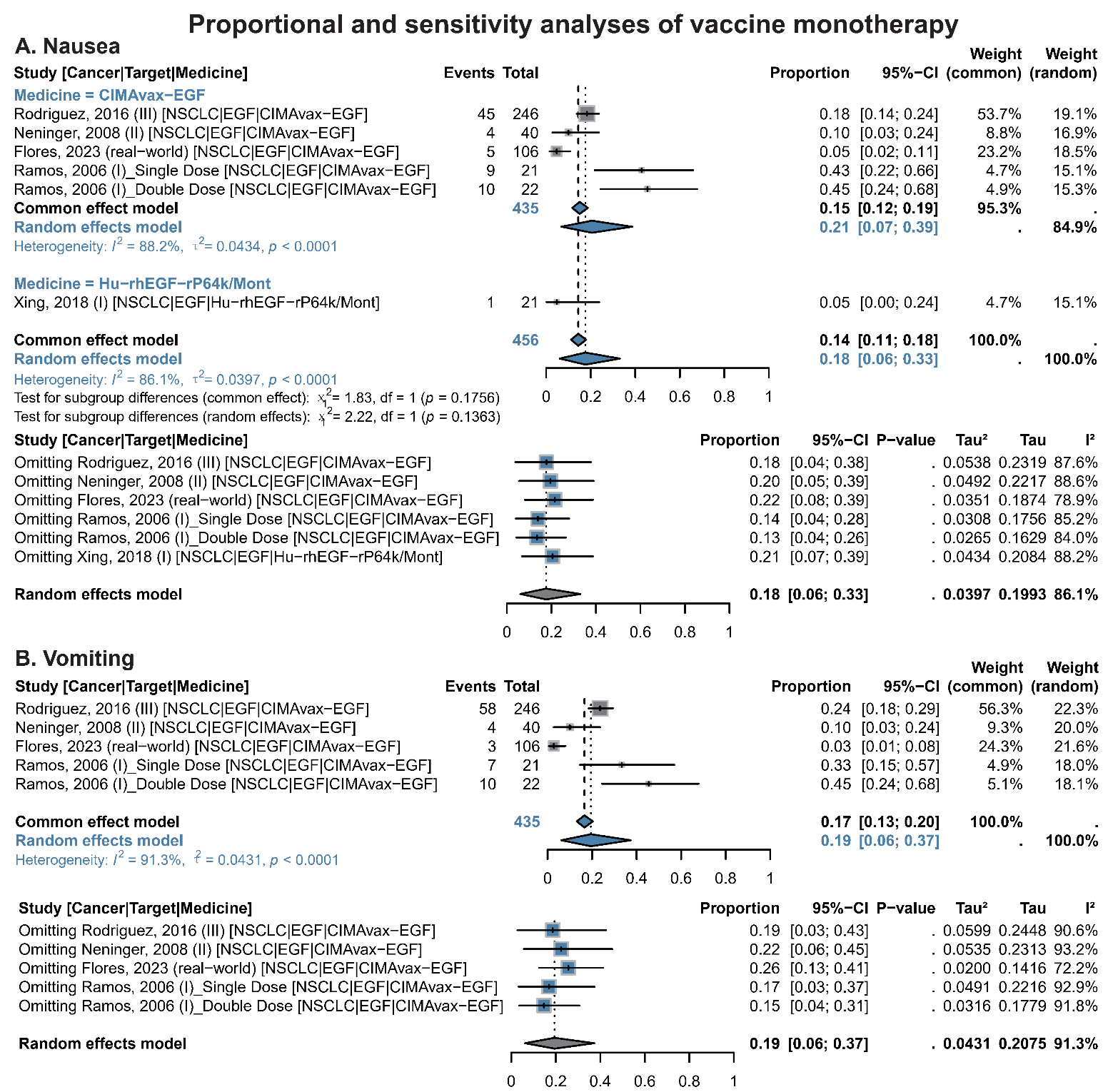 |
| --- |
| **Figure S19.** Proportional and sensitivity analyses of gastrointestinal symptoms incidence rates of EGF vaccines monotherapy, stratified by medicine type. (A) Nausea. (B) Vomiting. CI, confidence interval; EGF, epidermal growth factor; NSCLC, non-small cell lung cancer. Gray squares = individual study effect sizes (weighted by sample size). Blue diamonds = pooled effect sizes. Horizontal lines = 95% CI. In sensitivity analysis, blue squares = effect sizes after excluding one individual study; gray diamonds = original overall pooled effect sizes. |
